# *In silico* Analysis of Polymorphisms in microRNAs Deregulated in Alzheimer Disease

**DOI:** 10.3389/fnins.2021.631852

**Published:** 2021-03-24

**Authors:** Mahta Moraghebi, Reza Maleki, Mohsen Ahmadi, Ahmad Agha Negahi, Hossein Abbasi, Pegah Mousavi

**Affiliations:** ^1^Student Research Committee, Faculty of Medicine, Hormozgan University of Medical Sciences, Bandar Abbas, Iran; ^2^Student Research Committee, Department of Clinical Biochemistry, School of Medicine, Shahid Beheshti University of Medical Sciences, Tehran, Iran; ^3^Department of Internal Medicine, Faculty of Medicine, Hormozgan University of Medical Sciences, Bandar Abbas, Iran; ^4^Student Research Committee, Faculty of Para-Medicine, Hormozgan University of Medical Sciences, Bandar Abbas, Iran; ^5^Department of Medical Genetics, Faculty of Medicine, Hormozgan University of Medical Sciences, Bandar Abbas, Iran; ^6^Molecular Medicine Research Center, Hormozgan Health Institute, Hormozgan University of Medical Sciences, Bandar Abbas, Iran

**Keywords:** microRNA, miRNA, polymorphism, SNP, RNA-bindig proteins, RBP, Alzhaimer’s disease

## Abstract

**Background:**

Alzheimer’s disease (AD) is a degenerative condition characterized by progressive cognitive impairment and dementia. Findings have revolutionized current knowledge of miRNA in the neurological conditions. Two regulatory mechanisms determine the level of mature miRNA expression; one is miRNA precursor processing, and the other is gene expression regulation by transcription factors. This study is allocated to the in-silico investigation of miRNA’s SNPs and their effect on other cell mechanisms.

**Methods:**

We used databases which annotate the functional effect of SNPs on mRNA-miRNA and miRNA-RBP interaction. Also, we investigated SNPs which are located on the promoter or UTR region.

**Results:**

miRNA SNP3.0 database indicated several SNPs in miR-339 and miR-34a in the upstream and downstream of pre-miRNA and mature miRNAs. While, for some miRNAs miR-124, and miR-125, no polymorphism was observed, and also miR-101 with ΔG -3.1 and mir-328 with ΔG 5.8 had the highest and lowest potencies to produce mature microRNA. SNP2TFBS web-server presented several SNPs which altered the Transcription Factor Binding Sites (TFBS) or generated novel TFBS in the promoter regions of related miRNA. At last, RBP-Var database provided a list of SNPs which alter miRNA-RBP interaction pattern and can also influence other miRNAs’ expression.

**Discussion:**

The results indicated that SNPs microRNA affects both miRNA function and miRNA expression. Our study expands molecular insight into how SNPs in different parts of miRNA, including the regulatory (promoter), the precursor (pre-miRNA), functional regions (seed region of mature miRNA), and RBP-binding motifs, which theoretically may be correlated to the Alzheimer’s disease.

## Introduction

Alzheimer’s disease (AD) is a chronic neurodegenerative disease which slowly develops and worsens during the time. This disease manifests itself in the gradual and progressive loss of consciousness and memory. Currently, the prevalence of Alzheimer’s disease among middle-aged people in developed countries is about 5.1% (Mirzaii-Fini et al., 2018). Increasing life expectancy has led to an increase in people over the age of 60 in the world, as well as an increase in the prevalence of neurological diseases such as dementia. Based on a 2015 Alzheimer’s report, it is projected to reach more than 130 million people in the world by 2050 (Podhorna et al., 2020).

miRNAs, short double-stranded RNAs (dsRNA) about 18-24 nucleotides in length, negatively regulate the gene expression by direct binding 3′-untranslated region (UTR) of target messenger RNA (mRNA) and reduce its stability and translatability. This process is governed by the seed region (positions among 2nd-8th in miRNA) of miRNA (John et al., 2004). Several miRNAs have function in various processes including cell proliferation, cell death, lipid metabolism, neural pattern, hematopoietic differentiation, and immunity (Wahid et al., 2010). In recent years, studies have focused on the role of microRNAs in the complex diseases such as neurodegenerative diseases (Femminella et al., 2015). Several miRNAs regulate the genes which involved in the development of Alzheimer’s disease (Reddy et al., 2017).

**FIGURE 1:**
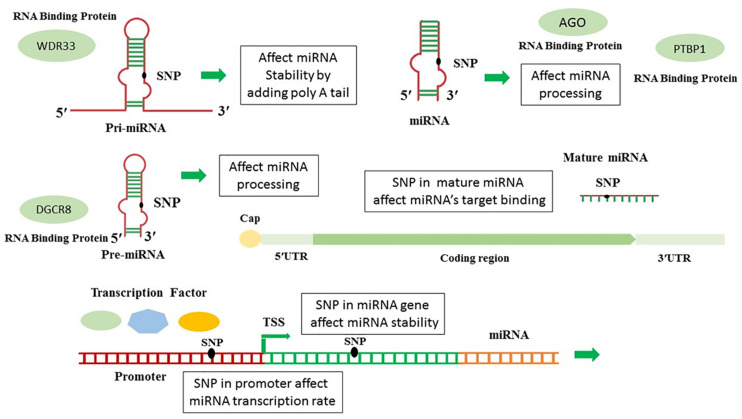
The ways that SNPs affect microRNAs expression and function.

The seed sequence binding to the target occurs in various ways which can be complete or incomplete (Witkos et al., 2011). Since miRNAs are small functional units, a single base change in both precursor blocks, as well as the mature miRNA sequence, may affect microRNAs evolution resulting in producing novel miRNA by different biological functions (Dong et al., 2013). Mutation in pri or pre-miRNA may affect the stability or processing of miRNA or mRNA. Mutation in the pri-mRNA or Cisor trans promoter may affect mature miRNAs’ transcription rate (Georges et al., 2007). The presence of SNPs in the miRNA’s seed regions is considerably influenced the miRNA’s target loss and gain (generates a novel repertoire of target genes); thus, altering the miRNA biological function significantly (Xu et al., 2013; Zhang Y. et al., 2019). Transcription factors (TFs) are the fundamental regulators of biological mechanisms which bind to transcriptional regulatory motifs (e.g., promoters, enhancers) to regulate their target genes’ expression in a sequence-specific manner (Lambert et al., 2018). Since the interaction of TFs and TF binding sites is integrated into gene regulatory systems, the variations at the TF or binding site alter this interaction and may lead to increasing or reducing the number of TFs by specific binding preferences; ultimately, impaired gene expression (Buroker et al., 2015). The biogenesis and maturation pathway of miRNA is a highly regulated mechanism. RNA-binding proteins (RBPs) are potent effectors which play a significant role in optimal miRNA biogenesis and function pathways in several sequential steps, including their efficient precursor’s processing, transfer, subcellular location, degradation, and biological activity and specificity (Van Kouwenhove et al., 2011; Treiber et al., 2017). SNPs may affect RBP-mediated post-transcriptional regulatory processes of gene expression via several mechanisms, including altering miRNA-target interaction, secondary RNA structure stability, and RBP-miRNA interplay (Figure 1; Mao et al., 2016; Treiber et al., 2017). SNPs located on the gene or its promoter, and these SNPs can also be associated to some diseases (Boutz et al., 2007; Delay et al., 2011; Roy and Mallick, 2017).

This study aims to investigate in-silico analysis of SNPs in miRNAs which control the genes involved in Alzheimer’s disease and possibly damage neuronal cells. For this purpose, we computationally evaluated the functional effect of polymorphisms in these miRNAs controlling the neurodegenerative function. The results may be useful to determine candidate SNPs for further functional analyzing and investigating causal SNPs underlying Alzheimer’s and developing hypotheses and testing to develop Alzheimer’s treatments.

## Materials and Methods

### Selection of miRNAs That Involve in Alzheimer

Hormozgan University of Medical Science’s ethics committee approved this research (ethical code: IR/HUMS.REC.270). Upstream miRNAs of genes directly involved in Alzheimer’s disease has been gained from recent review article and other major joints. In this study, PubMed, Embase, ScienceDirect, Cochrane Library, and Google Scholar databases were reviewed. Relevant keywords including microRNA, miRNA, AND Alzheimer’s disease, were used applying Medical Subject Heading (MeSH); finally we selected the articles to investigate the relationship among these microRNAs in Alzheimer’s disease. These miRNAs are recognized to be associated to Alzheimer’s disease and neurodegeneration.

### miRNA Involvement in the Pathogenesis of AD

To check which miRNAs are connected in AD’s pathogenesis, we used Human Disease MicroRNA Database 3.0 (HMDD v3.0)^1^, as a curated database which considers experiment-supported data for microRNA linkages and human disease, and we labeled them for connecting to Alzheimer’s diseases.

### *In silico* Prediction of SNPs Occurring in miRNA Genes

The website of An-Yuan Guo’s bioinformatics Lab^2^ has provided numerous databases for in silico studies. The tone of most important parts of this site is miRNASNPV3^3^, which makes it possible to check the potential effect of SNPs in miRNA maturation and function. miRNASNP includes SNPs in pre-miRNAs of human and other species, target gain and loss by SNPs in miRNA seed regions or 3′UTR of target mRNAs (Xie et al., 2020).

### *In silico* Investigation of SNPs Occurring in miRNA Promoter Genes

In this study, all microRNA promoters which involved in Alzheimer’s disease, were extracted. Ensemble (with genome assembly GRCh38.p13)^4^ was used to identify the promoter areas of microRNAs. Obtained areas were checked at the UCSC^5^ site, and all SNPs in promoter area were retrieved from database. SNP2TFBS web-server^6, 7^, was performed to analyze the functional effect of SNPs in transcription factor binding (TFB) affinity patterns (Treiber et al., 2017). It is in the Human genome assembly GrCH37/hg1 from the curated JASPAR CORE 2014 vertebrate motif database through Position Weight Matrix (PWM) calculation. We used the SNPViewer tool, a web-service that employs its rsID identifier to search for SNPs to identify changes altering the transcription factor binding areas (Treiber et al., 2017).

### *In silico* Investigation Impact of miRNAs SNPs on Their Interaction With RNA Binding Proteins and Expression of Other miRNAs

In this section, the RBP-Var database^8^ was employed to annotate the functional effect of SNPs on RNA binding protein affinity pattern and post-transcriptional interaction and regulation of miRNA, including its maturation, transportation from the nucleus to cytoplasm, and function. The data source for RBP-Var database was provided from starBase, CLIPdb, GEO, CISBP-RNA, RBPDB, dbSNP v142, RADAR, DARNED, TargetScan, miRanda, miRNASNP, MuTher, SCAN, seeQTL, GTEx, Harvard, and dsQTL Browser (Mao et al., 2016). All SNPs occurring in the miRNA gene (related to pri-miRNA, pre-miRNA, mature miRNA) were considered and uploaded to search box related to dbSNP. Finally, for determining and characterizing the conserved cis-motifs of RBP-RNA interaction (motif matches) in the transcriptome, RBP-Var uses all positional weight matrices of two databases, CISBP-RNA and RBPDB in AURA database. In this way, all potential k-mers are aligned with the transcriptome employing MAST in the MEME suite, a motif discovery algorithm, to present the final motif mapping with its default parameters, a match score > 0, and *p*-Values < 0.0001 (Mao et al., 2016).

### *In silico* Investigation of miRNAs’ SNPs on GWAS Catalog

genome-wide association study (GWA study, or GWAS), also known as whole-genome association study (WGA study, or WGAS), is a kind of study observant genome-wide set of genetic variants in different individuals whether the variant is associated to the trait. It is a study that looks at different genetic variants throughout the genome and examines in different individuals whether the variant is related to the trait. GWAS analysis typically focuses on associations between SNPs and traits, for example, major human diseases. The GWAS catalog is a freely available database that has collected genome-wide association studies (GWAS), summarizing unorganized data from different literature sources into accessible data. It has been a joint project between NHGRI and the European Bioinformatics Institute (EBI) since 2015 (MacArthur et al., 2017). We used miRSNPV3 (see text footnote 3), the “Disease” section. In the “Disease” module, the site integrated pathological information SNPs from the NHGRI GWAS catalog. For variations in miRNAs, the database provided the minimum free energy change of the pre-miRNAs secondary.

## Results

In this study, dysregulated microRNAs and their targets were collected. PubMed, Embase, ScienceDirect, Cochrane Library, and Google Scholar databases were reviewed. 38 dysregulated microRNAs and their targets were collected. Basic information for these microRNAs, including precursor ID, accession number, Genome position, host gene, mature miRNA showed in Supplementary Table 1 (It is provided in the supplementary). List of microRNAs, tissue type, their target genes, and microRNAs expression level were presented in Table 1. The miRNAs involvement in the pathogenesis of AD was tagged with ^∗^.

**TABLE 1:** List of miRNAs target genes correlated with Alzheimer disease.

microRNA	Tissue	Target	Expression	References
miR-101-2	_	*COX2, APP*	Downregulation	Vilardo et al., 2010; Delay et al., 2011
miR-103	Plasma	PTGS2	Downregulation	Wang et al., 2020
miR-106	-	Rb1, p73, p62	Downregulation	Delay et al., 2011
;miR-107*	Brain	CDK5R1	Downregulation	Moncini et al., 2017
	-	*BACE1, Cofilin, CDK6, Dicer*	Downregulation	Delay et al., 2011; Chen et al., 2020; Wang et al., 2020
miR-108	-	*ATM*	Downregulation	Delay et al., 2011
miR-1229	-	*SORL1*	-	Ghanbari et al., 2016
miR-124*	Brain	*BACE1*	Downregulation	Fang et al., 2012
miR-125	Brain	*DUSP6, PPP1CA, Bcl-W*	Upregulation	Banzhaf-Strathmann et al., 2014; Zhou et al., 2020
miR-126	Brain	*IRS-1 and PIK3R2*	Upregulation	Kim et al., 2016
miR-128*	Brain	*Aβ*	Upregulation	Tiribuzi et al., 2014
miR-130b	Cell culture	*p63*	Upregulation	Zhang R. et al., 2014
miR-132*	Brain	*PTEN, FOXO3a and P300*	Downregulation	Wong et al., 2013
	Frontal cortex	*sirt1*	Downregulation	Weinberg et al., 2015
miR-135	Peripheral blood	*BACE1*	Downregulation	Zhang Y. et al., 2016; Yang et al., 2018
miR-137*	Brain	*SPTLC1*	Downregulation	Geekiyanage and Chan, 2011
miR-146	CSF	*RNU44, RNU6b*	Downregulation	Muller et al., 2014; Lukiw, 2020
miR-15	Brain, hippocampus	*CDK5R1, ROCK1*	Downregulation	Moncini et al., 2017; Li X. et al., 2020
	_	*Bcl-2, ERK-1*	Downregulation	Delay et al., 2011
miR-16*	Neuronal cells	*APP*	Downregulation	Zhang et al., 2015
miR-181	Brain	*SPTLC1*	Downregulation	Geekiyanage and Chan, 2011
miR-188	Brain	*BACE1*	Downregulation	Guo et al., 2014; Zhang R. et al., 2014
miR-193*	Hippocampus	*APP*	Downregulation	Zhang R. et al., 2014; Yang et al., 2018
	Cell culture	*MAPK pathway*	Upregulation	Zhang R. et al., 2014
miR-20a*	Cell culture	*Bcl-2, MEF2D,MAP3K12*	Upregulation	Zhang et al., 2015
miR-200*	Plasma, hippocampus	*PRKACB*	Downregulation	Wang et al., 2019
miR-206*	Brain	*BDNF*	Upregulation	Tian et al., 2014
miR-212*	Frontal cortex	*sirt1*	Downregulation	Weinberg et al., 2015
	Brain	*PTEN, FOXO3a, P300*	Downregulation	Wong et al., 2013
miR-219*	Brain	*tau*	Downregulation	Santa-Maria et al., 2015
miR-23	Frontal cortex	*sirt1*	Downregulation	Weinberg et al., 2015
miR-26b*	Brain cortex	*Rb1*	Upregulation	Absalon et al., 2013
miR-29	Brain	*hBACE1*	Downregulation	Pereira et al., 2016
	-	*BIM, BMF, HRK, Puma*	Downregulation	Delay et al., 2011
Mir-29c*	Peripheral blood	*BACE1*	Downregulation	Yang et al., 2015
miR-298	Transgenic animals	*BACE1*	Downregulation	Boissonneault et al., 2009
miR-30	-	*BDNF*	-	Croce et al., 2013; Li L. et al., 2020
miR-33	-	*ABCA1*	-	Kim et al., 2015
miR-339	Brain	*BACE1*	Downregulation	Long et al., 2014
miR-34	-	*tau*	-	Dickson et al., 2013
	Brain	*VAMP2, SYT1, HCN1, NR2A, GLUR1, NDUFC2*	Upregulation	Sarkar et al., 2016
miR-328	Transgenic animals	*BACE1*	Downregulation	Boissonneault et al., 2009
miR-329	Cell culture	*Mef2*	Upregulation	Zhang R. et al., 2014
miR-603	Hippocampus	*LRPAP1*	Upregulation	Zhang C. et al., 2016
miR-9	CSN	*SIRT1*	Upregulation	Sethi and Lukiw, 2009; Souza et al., 2020

COX2, Cyclooxygenase 2; APP, Amyloid Beta Precursor Protein; Rb1, Retinoblastoma; BACE1, Beta-Secretase 1; CDK6, Cyclin Dependent Kinase 6; CDK5R1, Cyclin-dependent kinase 5 activator 1; ATM, Ataxia telangiectasia mutated; SORL1, Sortilin Related Receptor 1; DUSP6, Dual specificity phosphatase 6; IRS-1, Insulin receptor substrate 1; BDNF, Brain-derived neurotrophic factor; PPP1CA, Protein Phosphatase 1 Catalytic Subunit Alpha; sirt1, Sirtuin 1; FOXO3a, Forkhead Box O3; PIK3R2, Phosphoinositide-3-Kinase Regulatory Subunit 2; PTEN, Phosphatase and tensin homolog; RNU44, Small Nucleolar RNA, C/D Box 44; SPTLC1, Serine Palmitoyltransferase Long Chain Base Subunit 1; RNU6b, U6 Small Nuclear 6; Bcl-2, B-cell lymphoma 2; ERK-1, Extracellular Signal-Regulated Kinase; MEF2D, myocyte enhancer factor 2D; MAP3K12, Mitogen-Activated Protein Kinase Kinase Kinase 12; ABCA1, ATP Binding Cassette Subfamily A Member 1; BMF, Bcl2 Modifying Factor; Puma, P53 Up-Regulated Modulator Of Apoptosis; NDUFC2, NADH, Ubiquinone Oxidoreductase Subunit C2; BIM, Bcl-2-Related Ovarian Death Agonist; VAMP2, vesicle-associated membrane protein; HCN1, Hyperpolarization Activated Cyclic Nucleotide Gated Potassium Channel 1; HRK, Harakiri, BCL2 Interacting Protein; NR2A, N-methyl D-aspartate 2A; SYT1, Synaptotagmin 1; PTGS2, Prostaglandin-Endoperoxide Synthase 2; PRKACB, Protein Kinase CAMP-Activated Catalytic Subunit Beta; Mef2, Myocyte Enhancer Factor 2C; LRPAP1, Low density lipoprotein receptor-related protein-associated protein 1. *miRNA involved in the pathogenesis of AD.

### *In silico* Prediction and Functional Annotation of SNPs Occurring in miRNA Genes

In the next step, SNPs in miRNA genes were computationally analyzed. The miRNA SNPV3.0, the database of SNPs in miRNA was used to search SNPs of miRNAs. The server performs the prediction of miRNA target loss and gains through two target prediction tools, TargetScan, and miRmap. If one target gene of miRNA for wild type allele shows in both servers, but not in the mutant allele were considered the miRNA lost this target gene. On the contrary, if one target gene for mutant allele is shown in both servers, but not in wild type of allele, SNP-bearing mutant miRNAs achieve a target gene. The analysis of variant’s functional effect on pre-miRNA processing (for mature miRNA production) was performed through ΔG calculation which was the difference between minimal free energy (MFE), predicted by RNAfold online server, of wild type and SNP- miRNA. Moreover, we showed the exact location of SNPs and alternative alleles. The position of SNPs is indicated by Pre-miRNA, mature miRNA, or seed sequence. Results revealed several SNPs in pre-miRNA, mature miRNA, and seed site as indicated in Table 2. miR-339 and miR-34a have the majority of polymorphisms in the upstream and downstream of pre-miRNA and mature miRNAs, respectively, whereas some miRNAs have no SNPs, e.g., miR-124, and miR-125. A variant in miR-101-2 (rs138231885) has the most negative ΔG (−3.1) with a high expression rate of mature miRNA, while another SNPs (rs188892061) in miR-328 has the most ΔG (5.8) with a low expression rate of mature miRNA. The results of its investigation are given in Table 2.

**TABLE 2:** Data collected from miRNASNPv3, it shows microRNAs SNP, frequent, its position, allele, region and enthalpy.

pre-miRNA	SNP ID	Position	Ref/Alt	Region	ΔG	Predicted effect on mature miRNA expression
miR-101-2	rs138231885	chr9:4850301	T/C	pre-miRNA	−3.1	up
miR-106b	rs72631827	chr7: 99691652	C/A	pre-miRNA	0	mild
miR-107	rs199975460	chr10: 91352545	T/C	pre-miRNA	−0.7	mild
miR-1229-3p	rs200647784	chr5: 179225292	T/C	in_mature	−0.3	mild
miR-1229-3p	rs2291418	chr5: 179225324	G/A	in_mature	0	mild
miR-126	rs199992070	chr9: 139565134	C/T	pre-miRNA	3	down
hsa-miR-128-1-5p	rs117812383	chr2: 136422988	G/A	pre-miRNA	2.7	down
miR-130b	rs72631822	chr22: 22007634	G/A	pre-miRNA	−1	mild
miR-130b	rs140403670	chr22: 22007661	G/A	in_mature	3.9	down
miR-132	rs551930279	chr17:2050002	G/T	pre-miRNA	0	mild
miR-132	rs551930279	chr17:2050003	G/A	pre-miRNA	0	mild
miR-135b	rs573530355	chr1:205448310	C/G	pre-miRNA	0.8	mild
miR-135b	rs139405984	chr1: 205417483	C/G	pre-miRNA	0	mild
miR-135b	rs139405984	chr1: 205417483	C/T	pre-miRNA	0	mild
miR-146a	rs76149940	chr10: 104196269	C/T	pre-miRNA	1.9	mild
miR-146b	rs201978234	chr10: 102436580	C/A	pre-miRNA	2.9	down
miR-146b	rs201978234	chr10: 102436580	C/T	pre-miRNA	2.9	down
hsa-mir-16-1	rs371922256	chr13:50048974	T/C	pre-miRNA	0.6	mild
hsa-mir-16-1	rs72631826	chr13:50049007	A/G	pre-miRNA	0.5	mild
hsa-mir-16-1	rs72631826	chr13: 50623143	A/G	pre-miRNA	0.5	mild
miR-188	rs186369276	chrX: 50003535	G/T	in_mature	4.9	down
hsa-miR-188-3p	rs191840972	chrX: 49768168	C/T	in_seed	2.5	down
miR-193	rs60406007	chr17:31560014	G/T	pre-miRNA	4	down
miR-20a	rs185831554	chr13: 91351102	T/G	pre-miRNA	0.2	mild
miR-212	rs539716752	chr17:2050380	G/T	pre-miRNA	0.9	mild
miR23b	rs201848546	chr9: 95085213	G/A	pre-miRNA	4.2	down
miR-26b	rs565919718	chr2:218402647	C/T	pre-miRNA	2.2	down
miR-26b	rs188612260	chr2:218402684	C/T	pre-miRNA	0	mild
miR-298	rs201036298	chr20: 58818294	T/G	in_mature	3.4	down
miR-30a	rs149150037	chr6: 71403567	G/A	in_mature	1.6	mild
miR-30a	rs149150037	chr6: 71403567	G/C	in_mature	1.6	mild
miR-30a	rs190842689	chr6: 71403603	C/A	in_mature	3	down
miR-30a	rs190842689	chr6: 71403603	C/G	in_mature	3	down
miR-30a	rs190842689	chr6: 71403603	C/T	in_mature	3	down
miR-328	rs188892061	chr16: 67202389	C/A	Mature	5.8	down
miR-328	rs188892061	chr16: 67202389	C/T	Mature	5.8	down
miR-328	rs188892061	chr16: 67202389	C/G	Mature	3.10	down
miR-329	rs34557733	chr14: 101026792	G/GA	pre-miRNA	1.9	mild
miR-329	rs201061298	chr14: 101493169	G/A	pre-miRNA	2.7	down
miR-329-2	rs377234552	chr14:101027141	T/C	pre-miRNA	0	mild
miR-329-2	rs377234552	chr14:101027141	T/A	pre-miRNA	0	mild
miR-33	rs77809319	chr22: 41900991	A/G	in_seed	0	mild
miR-339	rs72631831	chr7: 1023020	C/T	pre-miRNA	−0.7	mild
miR-339	rs72631820	chr7: 1022963	T/C	in_mature	0.6	mild
miR-339	rs145196722	chr7: 1022990	C/T	in_mature	−0.7	mild
miR-339	rs72631831	chr7: 1023020	C/T	pre-miRNA	−0.7	mild
miR-339-5p	rs567174785	chr7:1023017	G/A	pre-miRNA	1.6	mild
miR-34a	rs201359809	chr1: 9151688	C/G	pre-miRNA	3.5	down
miR-34a	rs72631823	chr1: 9151723	C/T	pre-miRNA	0.87	mild
miR-34a	rs35301225	chr1: 9151743	C/T	in_mature	4.8	down
miR-34a	rs35301225	chr1: 9151743	C/A	in_mature	4.7	down
miR-603	rs11014002	chr10:24275724	C/T	pre-miRNA	−1.8	mild
miR-603	rs11014002	chr10:24275724	C/A	pre-miRNA	0	mild

*Finally, the effect of SNP on microRNA expression is shown.ΔG, The difference of MFE between wild type allele and mutant allele. Underlined SNPs have linkage disequilibrium.*

### *In silico* Investigation of SNPs Occurring in miRNA Promoter Genes

SNPs’ impact was investigated in the promoter regions of miRNAs which target genes directly involved in Alzheimer’s disease. Putative TF binding sites from human genome assembly GrCH37/hg1 (for wild type allele) and 1000 Genomes project (for a mutant allele with MAF ≥ 0.001) which merged, were calculated through Position Weight Matrix (PWM) calculation (PWM score) from the curated JASPAR CORE 2014 vertebrate motif database. These SNPs affect the transcription level of miRNAs which can be increased, decreased, or neutralized. The location of SNPs, their specific numbers, and their effect are given in Table 3. As shown in Table 3, some miRNAs have several promoter regions, each of which has multiple SNPs. Nevertheless, not all of them affect expression.

**TABLE 3:** List of SNPs are located in the promoter region and their effect on transcription factor binding performed by SNP2TFBS web-server.

miRNA	Promoter regions	More PWM score on Alt (Scorediff +) missing in ref	More PWM score on Ref (Scorediff −) missing in alt	Neutral
miR-106b	Chromosome 7: 100,088,200-100,090,401	rs7807156	-	-
	Chromosome 7: 100,099,400-100,103,001	rs547370604, rs115396052, **rs2293481**	rs1122598	-
miR-1229-3p	Chromosome 5: 179,793,600-179,797,201	rs3756614	rs138686538	rs116280439
	Chromosome 5: 179,804,000-179	-	rs59108011	rs146231546, rs546034674, rs559539498, rs73351618
miR-124	Chromosome 8: 9,902,600-9,907,401	rs608095, rs77162181	-	rs558057975
miR-125	Chromosome 19: 51,687,200-51,693,001	rs112214384, rs71189613, rs62106945, rs543280604, rs192652956, rs8112073, rs8111799	rs10405559, rs72626247, rs77124947, rs149747756, rs139781159, rs117342253, rs73934279, rs78367065, rs882105, rs35627212, rs141394647, rs138807245	rs78241354, rs59801018
	Chromosome 19: 51,701,600-51,705,801	rs73054887	rs2305373, rs145355379, rs370152118, rs73054887	rs2290282
miR-126	Chromosome 9: 136,655,800-136,671,201	rs4880116, rs78431904, rs143084454, rs74973741, rs73668352, rs143871100, rs114709635	rs74557797, rs4880116, rs9411259, rs4880062, rs74722250, rs944753, rs75759763, rs13297806, rs12375984, rs111978941, rs28758526, rs2297535, rs1140713	rs78549582, rs76530857, rs78785680, rs78431904, rs200025885, rs4880118,
miR-128	Chromosome 2: 135,663,601-135,667,799	rs17652559	rs139103196, rs2034276	rs200284798
miR-130b	Chromosome 22: 21,650,800-21,653,601	rs412596, rs373001	rs373001, rs861843	rs3804071
	Chromosome 22: 21,657,000-21,659,001	rs138259296, rs34932470	rs384262	rs114526180, rs116782856
miR-137	Chromosome 1: 98,042,601-98,050,001	rs116048198, rs12744323, rs112984663, rs78422095, rs141931471, rs61786697	rs112693582, rs552418648	rs369374378
	Chromosome 1: 98,052,800-98,055,401	rs2660302	rs72969637	-
miR-146	Chromosome 5: 160,478,800-160,479,001	-	-	-
miR-193b	Chromosome 17: 31,558,001-31,562,401	rs75259244	rs74987923, rs74987923, rs73991207, rs56908712	rs71697208
	Chromosome 17: 31,565,000-31,565,401	rs118043603	-	-
	Chromosome 17: 31,567,000-31,567,201	-	-	-
miR-20a	Chromosome 13: 91,346,401-91,351,201	rs143640687	rs138151712, rs10630963, rs4284505	rs1888138 rs2351704
	Chromosome 13: 91,351,400-91,351,601	-	-	-
miR-26b	Chromosome 2: 218,394,800-218,402,201	rs2279014, rs2739047, rs149904564, rs115942360	rs73990437, rs116233374, rs116783631, rs186575073	rs1809231 rs10189062 rs3795985
miR-339-5p	Chromosome 7: 1,026,800-1,029,601	-	rs74360401, rs4074129 rs80224080	rs71020558
	Chromosome 7: 1,029,800-1,030,001	-	-	-
miR-328	Chromosome 16: 67,191,200-67,194,001	rs3730395	-	-
	Chromosome 16: 67,198,400-67,200,600	-	rs115994559, rs8059662	-
miR-9	Chromosome 1: 156,417,001-156,417,801	-	-	-
	ChromosoC12:H38me 1: 156,418,800-156,422,201	rs528893347, rs112487499, rs184035466	-	-

*Ref = The allele in the reference genome. Alt = Any other allele found at that locus. PMW = position weight matrices, a positive score implies a higher PWM score in the alternate allele. The underlined and bolded rsSNP is Expression quantitative trait loci (eQTL), rs2293481, P-value: 0.000004, Tissue: Nerve Tibial, source: GTEx_V4 (Genotype-Tissue Expression (GTEx) consortium) (Sonawane et al., 2017). eQTLs are genomic loci that show variation in the expression amount of mRNA transcript or a protein. These are usually the production of a single gene located in a specific chromosome area. The chromosomal locations that explain the variance of expression traits are called eQTL. Expression quantitative trait loci (eQTLs) are genomic loci that show variation in the expression amount of mRNA transcript or a protein. These are usually the production of a single gene located in a specific chromosome area. The chromosomal locations that explain the variance of expression traits are called eQTL. As we have mentioned in Supplementary Table 1, all of this microRNA is located in the intronic or intergenic area; however, eQTL included mRNAs. Thus, as we have expected, all of this miRNA, except one, was not found in the eQTL database (Rockman and Kruglyak, 2006; West et al., 2007; Majewski and Pastinen, 2011).*

Scorediff column describes the difference in PWM scores between alternating (mutant) and reference (wild type) alleles. Hence, a positive score means a larger PWM score in the alternating allele.

SNPs are only listed in the table which may affect miRNAs expression through affecting transcription factor binding sites for the transcription factor to bind. The meaning of reference genome (Ref) is a wild type allele in the table, and the alternate genome (Alt) is a mutant allele.

### *In silico* Investigation Impact of miRNAs SNPs on Their Interaction With RNA Binding Proteins and Expression of Other miRNAs

**FIGURE 2:**
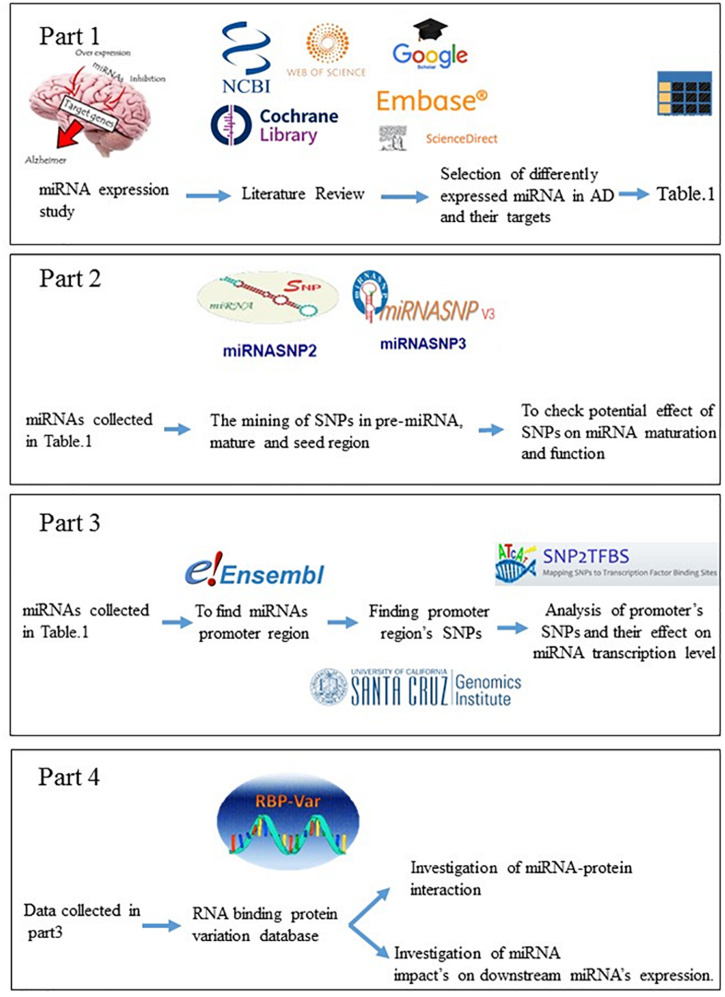
Graphical abstract, the methodology of study.

The interplay between RNA-binding proteins (RBPs) and miRNA together is considered as critical players to regulate many cellular processes of neuronal development and function (Hafner et al., 2010). The interaction between miRNAs and RNA-binding proteins is other issue which is affected by SNPs. As Table 4 shows, the most affected RNA binding proteins are the AGO family, PTBP1, WDR33, and DGCR8. Ago family are ubiquitously expressed which bind to miRNAs or siRNAs to guide post-transcriptional gene silencing either by destabilizing the mRNA or by translation repression (Höck and Meister, 2008). PTBP has a role in pre-mRNA splicing (Zhang et al., 2015), and WDR33 acts in 3′UTR polyadenylation (Chan et al., 2014). We investigated SNPs’ effect on other cell processes such as the maturation of microRNAs and their transfer to cell. The miRNAs sequences were scanned to identify conserved motifs of RBP-RNA interaction. Motifs discovered in RBPs-RNA and promoters by MEME Suite are shown in Table 5. Other salient point is considering the effect of microRNAs’ SNPs on the expression of another microRNA derived from the studying microRNAs which the results were shown in the Supplementary Table 2. This table contains the microRNA containing the SNPs and its effect (loss or gain) on the target microRNA and its *P*-value. All steps are summarized in Figure 2.

**TABLE 4:** Catalog of SNPs in miRNAs and their impact on miRNA- RNA Binding Protein interaction pattern provided by RBP-Var2 database.

miRNA’s Name	SNP’s Name	Chromosome location	RNA binding protein	RBP-Var score
miR-101-2	rs138231885	9:4850300-4850301	PTBP1, WDR33	2c
miR-106b	rs72631827	7:99691651-99691652	DGCR8, AGO2, AGO1, AGO3	β
miR-107	rs199975460	10:91352544-91352545	AGO	3α
miR-1229-3p	rs200647784	5:179225291-179225292	AGO1, AGO2	γ
miR-1229-3p	rs2291418	5:179225323-179225324	AGO1, AGO2	β
miR-128	rs117812383	2:136422987-136422988	AGO1, AGO2, AGO3, DGCR8	β
miR-130b	rs72631822	22:22007633-22007634	PTBP1	α
miR-130b	rs140403670	22:22007660-22007661	EIf4AIII, AGO, DGCR8, AGO2, FMR1, WDR33, AGO1, AGO3, AGO4, LIN28A, LIN28B	α
miR-135b	rs139405984	1:205417482-205417483	AGO2	β
miR-146b	rs76149940	13:50623142-50623143	PTBP1	α
miR-16	rs72631826	13:50623109-50623110	AGO1, AGO2, eIF4AIII, nSR100, PTBP1, nSR100	β
miR-16	rs72631826	X:49768140-49768141	AGO1, AGO2, eIF4AIII, nSR100, PTBP1, nSR100	β
miR-188	rs186369276	X:49768167-49768168	AGO1, AGO2, AGO3, AGO4, WDR33, FUS	β
miR-188	rs191840972	17:29887032-29887033	AGO1, AGO2, AGO3, WDR33	β
miR-193	rs60406007	13:92003355-92003356	DGCR8	β
miR-20a	rs185831554	9:97847494-97847495	DGCR8, AGO1, AGO2, AGO3, TIAL1, nsr100, LIN28B	α
miR23b	rs201848546	2:219267406-219267407	PTBP1, DGCR8	β
miR-26b	rs188612260	2:219267369-219267370	AGO2, DGCR8	β
miR-26b	rs565919718	20:57393348-57393349	AGO, DGCR8	α
miR-26b	rs188612260	6:72113269-72113270	DGCR8	β
miR-298	rs201036298	6:72113305-72113306	AGO3, PTBP1	β
miR-30a	rs149150037	22:42296994-42296995	AGO1, AGO2, AGO3, AGO4, DGCR8, WDR33, eIf4AIII	β
miR-30a	rs190842689	14:1062655-1062656	AGO, AGO1, AGO2, AGO3, AGO4, DGCR8, WDR33, LIN28A, eIF4AIII, PTBP1, FXR1, FMR1, FUS	β
miR-33	rs77809319	14:1062598- 1062599	AGO1, AGO2, AGO3, PTBP1, WDR33	β
miR-339	rs72631831	14:1062625-1062626	DGCR8	β
miR-339	rs72631820	14:1062652-1062653	AGO1, AGO2, AGO3, DGCR8, WDR33	α
miR-339	rs145196722	1:9211746-9211747	AGO1, AGO2, AGO3, DGCR8, WDR33, DGCR8	β
miR-339	rs567174785	1:9211801-9211802	DGCR8, WDR33	β
miR-34a	rs201359809	9:4850300-4850301	AGO2, DGCR8	β
miR-34a	rs72631823	7:99691651-99691652	AGO1, AGO2, DGCR8, nSR100	β
miR-34a	rs35301225	10:91352544-91352545	AGO1, AGO2, AGO3, AGO4, WDR33, nSR100, PTBP1, FUS, C22ORF28, FMR1	β

*AGO proteins (Argonaut) are ubiquitously expressed and bind to siRNAs or miRNAs to guide post-transcriptional gene silencing either by destabilization of the mRNA or by translational repression.DGCR8 microprocessor complex subunit (DiGeorge syndrome chromosomal region 8).PTBP1 Polypyrimidine tract-binding protein 1. Plays involves in pre-mRNA splicing and in the regulation of alternative splicing events.WDR33 Essential for both cleavage and polyadenylation of pre-mRNA 3′ ends.EIf4AIII ATP-dependent RNA helicase. Plays a role in pre-mRNA splicing as component of the spliceosome. FMR1 (fragile X mental retardation 1) Multifunctional polyribosome-associated RNA-binding protein.FXR1 (Fragile X mental retardation syndrome-related protein 1) regulate intracellular transport and local translation of certain mRNAs.LIN28A (Protein lin-28 homolog A) Inhibits the processing of pre-let-7 miRNAs and regulates translation of mRNAs.LIN28B (Protein lin-28 homolog B) Suppressor of microRNA (miRNA) biogenesis.nSR100 Splicing factor specifically required for neural cell differentiation. FUS DNA/RNA-binding protein that plays a role in various cellular processes such as transcription regulation, RNA splicing, RNA transport, DNA repair and damage response. Likely to affect RBP binding: α. Minimal possibility to affect RBP binding: β. Less likely to affect RBP binding: γ.*

**TABLE 5:** The sequence Logos (consensus sequences) in the RNA-Binding Protein motifs of miRNA via MEME analysis by RBP-Var2.

RBP Motifs	SNPID	Location	*P*_value	Score	Motifs
**In pre-miRNA, mature**					
**and seed region**					
miR-101-2	rs138231885	chr9: 4850298-4850305	0.000085	1070.750	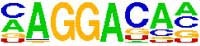 SRSF1_M106
miR-101-2	rs138231885	chr9: 4850298-4850305	0.000072	479.890	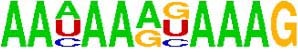 ZFP36L1_M269
miR-101-2	rs138231885	chr9: 4850298-4850305	0.000072	479.890	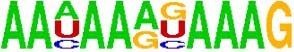 ZFP36L2_M269
miR-101-2	rs138231885	chr9: 4850298-4850305	0.000072	479.890	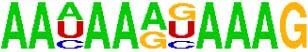 ZFP36L2_M269
miR-339	rs72631820	chr7:1062597-1062603	1086.880	0.000053	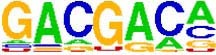 RBM45_M209
miR-107	rs199975460	chr10:91352539-91352546	1106.710	0.000085	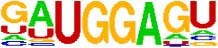 SF3B4_M205
miR-101-2	rs138231885	chr9:4850298-4850305	1070.750	0.000085	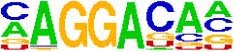 SRSF1_M106
miR-101-2	rs138231885	chr9:4850298-4850303	479.890	0.000072	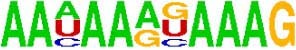 ZFP36L1_M269
miR-101-2	rs138231885	chr9:4850298-4850303	479.890	0.000072	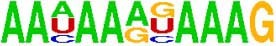 ZFP36L2_M269
miR-101-2	rs138231885	chr9:4850298-4850303	479.890	0.000072	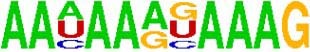 ZFP36_M269
miR-1229-3p	rs200647784	chr5:179225286-179225296	1477.030	0.000007	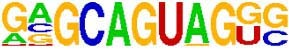 SNRPA_M347
**In Promoter region**					
miR-1229-3p	rs200647784	chr5:179225286-179225296	1477.030	0.000007	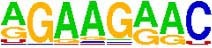 SNRPB2_M347
miR-106	rs115396052	chr7:99697034-99697041	1285.530	0.000018	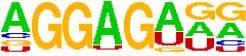 SRSF1_M272
miR-106	rs547370604	chr7:99697031-99697038	1112.400	0.000041	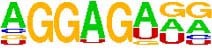 ENSG00000180771_M070
miR-106	rs547370604	chr7:99697031-99697038	1112.400	0.000041	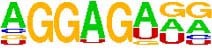 SRSF2_M070
miR-106	rs547370604	chr7:99697034-99697041	1285.530	0.000018	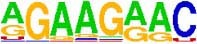 SRSF1_M272

### *In silico* Investigation of miRNAs’ SNPs on GWAS Catalog

All microRNAs regulated in Alzheimer’s disease were located in intergenic or intronic loci, none of which were found in the GWAS database. Moreover, some new SNPs in new microRNAs have been found. Although their expression has not been measured, they include some SNPs that can affect their regulation. Table 6 demonstrates that miR-4653 has the least amount of ΔG and the most effect on miR-4653 expression. On the contrary, miR-4698 has the most ΔG and the least impact on miR-4698 expression. GWAS catalog numbers also have been mention in Table 6.

**TABLE 6:** miRNAs and SNPs in Alzheimer’s GWAS catalog.

miRNAs	Mutation ID	Location	Ref/Alt	GWAS catalog	Region	ΔG	Predicted effect on expression
hsa-mir-324	rs200471575	chr17:7223379	G/C	Alzheimer’s disease with no specific cognitive domain impairment (PMID:30514930)	pre-miRNA	0	mild
hsa-mir-3622a	rs66683138	chr8:27701697	G/A	Alzheimer’s disease or family history of Alzheimer’s disease (PMID:29777097)	Mature	3.9	down
hsa-mir-1236	rs185147690	chr6:31956854	G/A	Alzheimer’s disease (PMID:30636644)	Seed	-3.6	up
hsa-mir-378i	rs9607855	chr22:41923272	C/T	Alzheimer’s disease (PMID:30636644)	Mature	0.4	mild
hsa-mir-4642	rs572524399	chr6:44435664	T/A	Alzheimer’s disease with visuospatial domain impairment (PMID:30514930)	Mature	1.4	mild
hsa-mir-4642	rs67182313	chr6:44435701	A/G	Alzheimer’s disease with visuospatial domain impairment (PMID:30514930) Alzheimer disease and age of onset (PMID:26830138)	pre-miRNA	-2.3	up
hsa-mir-4698	rs832733	chr12:47187846	T/A	Alzheimer’s disease (PMID:19118814)	pre-miRNA	4.2	down
hsa-mir-4698	rs185381854	chr12:47187856	T/G	Alzheimer’s disease (PMID:19118814)	pre-miRNA	4.2	down
hsa-mir-4487	rs539864281	chr11:47400994	G/C	Alzheimer’s disease or family history of Alzheimer’s disease (PMID:29777097)	pre-miRNA	6.2	down
hsa-mir-4658	rs142606351	chr7:100156636	G/A	Alzheimer’s disease or family history of Alzheimer’s disease (PMID:29777097)	pre-miRNA	0	mild
hsa-mir-4653	rs11983381	chr7:101159505	A/G	Alzheimer’s disease (PMID:30636644)	pre-miRNA	-5.1	up
hsa-mir-3908	rs111803974	chr12:123536470	C/T	Late-onset Alzheimer’s disease (PMID:27770636)	pre-miRNA	0	mild
hsa-mir-1229	rs2291418	chr5:179798324	G/A	Alzheimer’s disease (late onset) (PMID:24162737)	Mature	0	mild
hsa-mir-8086	rs11436116	chr10:28289300	CAA/C	Psychosis and Alzheimer’s disease (PMID:22005930)	pre-miRNA	0.2	mild
hsa-mir-5004	rs369274154	chr6:33438351	T/C	Late-onset Alzheimer’s disease (PMID:27770636)	Mature	1.7	mild
hsa-mir-8074	rs114948808	chr19:51206966	G/A	Alzheimer’s disease (PMID:18976728)	pre-miRNA	-0.1	mild
hsa-mir-8074	rs114948808	chr19:51206966	G/T	Alzheimer’s disease (PMID:18976728)	pre-miRNA	0	mild
hsa-mir-6503	rs545722613	chr11:60209147	G/A	Family history of Alzheimer’s disease; Alzheimer’s disease (late onset);Alzheimer’s disease or family history of Alzheimer’s disease (PMID:30617256) Alzheimer’s disease (late onset) (PMID:28714976)	pre-miRNA	0	mild
hsa-mir-633	rs17759989	chr17:62944250	A/G	Alzheimer’s disease with language domain impairment (PMID:30514930)	pre-miRNA	0.6	mild
hsa-mir-633	rs181392999	chr17:62944264	A/C	Alzheimer’s disease with language domain impairment (PMID:30514930)	pre-miRNA	-0.7	mild
hsa-mir-8084	rs404337	chr8:93029770	G/A	Logical memory (immediate recall) in Alzheimer’s disease dementia (PMID:29274321)	Mature	2.8	down
hsa-mir-492	rs200816308	chr12:94834403	A/C	Alzheimer’s disease (PMID:24755620)	pre-miRNA	0	mild
hsa-mir-6840	rs562470235	chr7:100356712	G/A	Alzheimer’s disease (late onset); Alzheimer’s disease or family history of Alzheimer’s disease (PMID:30617256)	Mature	1.3	mild
hsa-mir-4788	rs187884409	chr3:134437840	G/A	Late-onset Alzheimer’s disease (PMID:27770636)	Seed	3.8	down
hsa-mir-6892	rs6464546	chr7:143382713	G/A	Alzheimer’s disease or family history of Alzheimer’s disease (PMID:29777097)	pre-miRNA	-0.2	mild
hsa-mir-6892	rs6464546	chr7:143382713	G/C	Alzheimer’s disease or family history of Alzheimer’s disease (PMID:29777097)	pre-miRNA	-0.3	mild
hsa-mir-6892	rs150791328	chr7:143382732	C/T	Alzheimer’s disease or family history of Alzheimer’s disease (PMID:29777097) Alzheimer’s disease (late onset); Alzheimer’s disease or family history of Alzheimer’s disease (PMID:30617256) Alzheimer’s disease (late onset) (PMID:24162737) Alzheimer’s disease in APOE e4- carriers (PMID:25778476)	pre-miRNA	-0.3	mild
hsa-mir-8086	rs11436116	chr10:28289300	CAA/CAAA	Pulmonary function decline (PMID:22424883)	pre-miRNA	0.5	mild
hsa-mir-8086	rs11436116	chr10:28289300	CAA/CA	Psychosis and Alzheimer’s disease (PMID:22005930)	pre-miRNA	0.2	mild
hsa-mir-8485	rs551272692	chr2:50696214	A/G	Alzheimer’s disease with multiple cognitive domain impairments (PMID:30514930)	pre-miRNA	-0.4	mild
hsa-mir-8485	rs559970090	chr2:50696223	C/T	Alzheimer’s disease with multiple cognitive domain impairments (PMID:30514930)	pre-miRNA	0.9	mild
hsa-mir-8485	rs559970090	chr2:50696223	C/A	Alzheimer’s disease with multiple cognitive domain impairments (PMID:30514930)	pre-miRNA	0.9	mild
hsa-mir-8485	rs147396981	chr2:50696254	T/C	Alzheimer’s disease with multiple cognitive domain impairments (PMID:30514930)	pre-miRNA	-2.1	up

*ΔG: The difference of MFE between wild type allele and mutant allele.*

The underlined and bolded rsSNP is Expression quantitative trait loci (eQTL), rs2293481, *P*-value: 0.000004, Tissue: Nerve Tibial, source: GTEx_V4 (Genotype-Tissue Expression (GTEx) consortium) (Sonawane et al., 2017). eQTLs are genomic loci that show variation in the expression amount of mRNA transcript or a protein. These are usually the production of a single gene located in a specific chromosome area. The chromosomal locations that explain the variance of expression traits are called eQTL. Expression quantitative trait loci (eQTLs) are genomic loci that show variation in the expression amount of mRNA transcript or a protein. These are usually the production of a single gene located in a specific chromosome area. The chromosomal locations that explain the variance of expression traits are called eQTL. As we have mentioned in Supplementary Table 1, all of this microRNA is located in the intronic or intergenic area; however, eQTL included mRNAs. Thus, as we have expected, all of this miRNA, except one, was not found in the eQTL database (Rockman and Kruglyak, 2006; West et al., 2007; Majewski and Pastinen, 2011).

## Discussion

**FIGURE 3:**
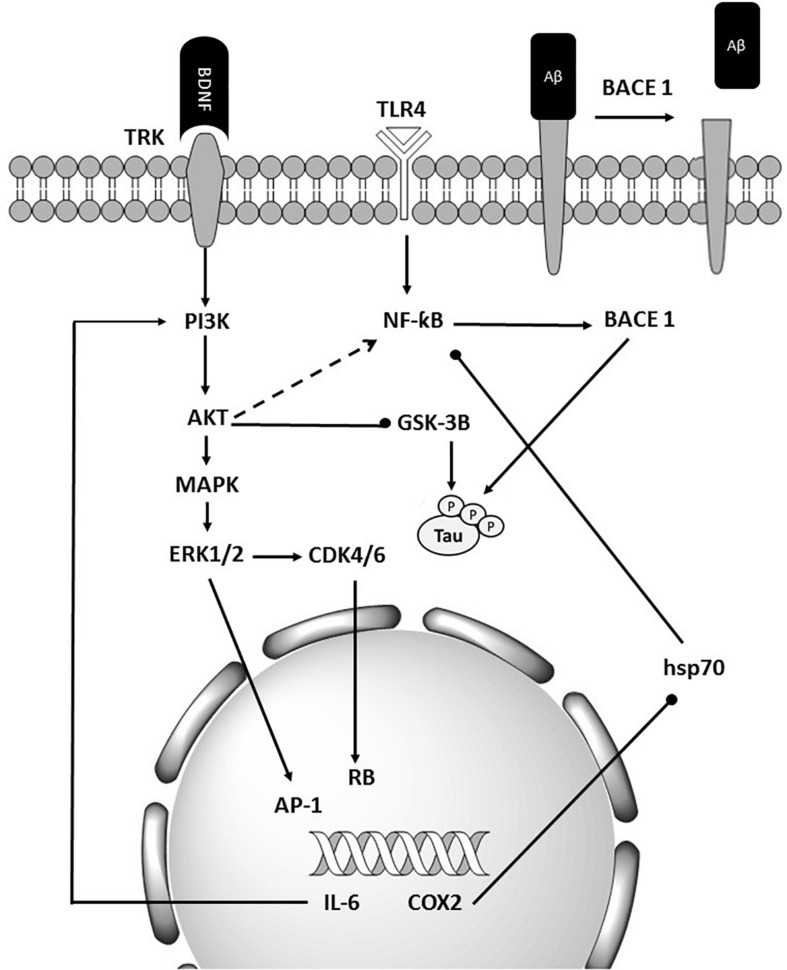
Schematic representation of BACE1’s importance in Alzheimer’s diseases. BACE1 is the final target of many miRNAs that are deregulated in Alzheimer’s disease. It also affected the tau and Aβ accumulation.

Given the level of information and advances in the bioinformatics, computational predictions of causal factors are served as a complementary strategy to facilitate the experimental characterization of multifactorial diseases. Although up to 92% of mammalian genes could be regulated by miRNA, only a few target pairs of miRNAs have been empirically analyzed (Boissonneault et al., 2009). Several problems including complexity, expensive, and overcome technical challenges such as tissue specificity, low expression, 3′ UTR selection, and miRNA stabilization, make current techniques a challenge for the experimental validation of relationships between miRNAs and their mRNA targets (Andrés-León et al., 2017). Identifying functional SNPs in genes and analyzing their effects on phenotypes may provide an opportunity for a more in-depth understanding of the potential impact of producing such alterations. SNPs in human miRNA genes influence biogenesis, expression level, and biological function. Impaired miRNA processing may generate isomiR which can change in Drosha and/or Dicer processing sites, leading to a complete change in downstream processes including the targeted mRNA transcripts, regulatory pathway, and complex phenotypes, and diseases (Starega-Roslan et al., 2015). Researchers have designed various efficient bioinformatics tools to annotate the potential effects of SNPs. All microRNAs involved in Alzheimer’s disease and their target genes were collected. Also, we briefly introduced theoretical methods to predict these functional SNPs. The results show that miR-298, miR-328, miR-124, miR-135b miR-188-3p, mir-29c, miR-339-5p, and miR-107 target the *BACE1* gene. Also, in 2009, Boissonneault et al. confirmed that dysfunctional interaction between miR-328 and BACE1 could be associated to Alzheimer’s disease.; Therefore, this gene plays a vital role in Alzheimer’s disease (Cole and Vassar, 2007; Boissonneault et al., 2009). Yan and Vassar (2014) have done a comprehensive search on BACE1 as a critical gene target for the therapy of Alzheimer’s disease. They asserted that β secretase, β-site amyloid precursor protein cleaving enzyme 1 (BACE1), launches producing toxic amyloid β (Aβ) through separating the extracellular domain of APP which plays a crucial role in Alzheimer’s disease pathogenesis (Yan and Vassar, 2014). In Alzheimer’s disease, amyloid bodies accumulate outside the neurons in some areas of brain and fibrous protein structures in the cell body of neurons, causing some changes in nerve cells’ proteome and disruption. One of the most critical proteins involved in Alzheimer’s disease is amyloid precursor protein (APP). APP protein, expressed in the nervous system cells, is involved in binding cells to each other, cell contact, and binding to the extracellular matrix and cytoskeleton. In addition, miR-101, miR-16, and miR-188 directly target APP gene (Vilardo et al., 2010; Zhang R. et al., 2014; Zhang et al., 2015). Three types of proteolytic enzymes could process APP protein, including BACE1, to form a peptide called amyloid-beta. Normally, the number of these fragments is small in the cells, and they quickly decompose; but if this balance is disturbed in the proteome of nerve cells and the amount of these components increases, spherical protein structures are formed, resulting in Alzheimer’s disease (Mullan et al., 1992; Zhang Y.W. et al., 2011; Jonsson et al., 2012). A 2019 study by Wang et al. on microRNAs involved in Alzheimer’s disease showed that the most common target was BACE1, or the direct target of BACE1, APP which underscores the importance of these genes (Mullan et al., 1992). Investigating other target genes in microRNAs has found that many of them, including the MAPK pathway, is the upstream of BACE1 and induce higher expression of BACE1 in its downstream (Figure 3; Kitagishi et al., 2014; Matsuda et al., 2018; Shal et al., 2018; Meng et al., 2020).

As the results show, the maximum number of polymorphisms was belonged to miR-339 in the upstream and downstream of mature regions in pre-miRNA and not within the seed region, while some microRNAs such as miR-124 and miR-125, there is no polymorphism in the pre-miRNA region. Imperatore et al. has declared that the level of miRNA-1229-3p which has been confirmed to regulate post-transcriptionally SORL1, is increased in the rs2291418 pre-miRNA-1229 variant. Using various biophysical techniques indicated that pre-miRNA-1229 normally forms a G-quadruplex structure in equilibrium with hairpin structure. The presence of this polymorphism, G/A, in pre-miRNA-1229 disturbs this balance (Imperatore et al., 2020).

Since interplay between miRNA and target mRNA is necessary for miRNA function, SNPs present on target binding sites of miRNAs should be evaluated before studies, especially gene expression.

Comparing ΔG (The difference of MFE between wild type allele and mutant allele) was shown in Table 2. According to the results, the highest ΔG related to miR-101 indicates the effect of T/C substitution which can increase the processing probability of pri-miRNA 101; thus, increase the production of its mature form.

According to the results of the highest ΔG in the miR-101, indicating the effect of T/C replacement can increase the processing; thus, it increases producing its mature form. According to the evidence, COX2, an inductive enzyme which catalyzes the conversion of arachidonic acid to prostanoids, plays a vital role in the plasticity of neurons and memory acquisition It seems that variant rs138231885, which is predicted to increase the expression of the mature form of miR-101-2 (performing biological function), is likely to be associated to disease risk.

The lowest number occurs in miR-328, miR-188, and miR-34. On the one hand, comparing Tables 1, 2 is shown that level expression of few microRNAs is different due to their mutations effect which could occur in them; for example, miR-101, miR-126, miR-128, miR-34a, miR-193, and miR-26; On the other hand, there are microRNAs in which effect mutations are in the same direction as their expression in Alzheimer’s disease. The miR-146a, miR-298, miR-30a, and miR-34a are from this category. Hu et al. suggested two common polymorphisms in pre-miR-125a may contribute to a genetic disorder called RPL with a disturbance in the miR-125a’s expression (Hu et al., 2011). Inoue et al. (2014) has found that miR-125 and its SNPs (rs12976445) have a negative relationship with Graves’ disease (GD) and Hashimoto’s disease (HD); moreover, not only the expression of miRNA-125 but also its efficacy has been reduced. Moreover, Landi et al. (2008) have investigated polymorphisms which have affected micro-RNA-binding sites and their attachment to targets.

The results of Table 3 provide the list of regulatory SNPs which significantly affect transcription factor binding sites for the transcription factor affinity. According to the evidence, variants placed in non-coding regions which may affect gene expression by changing the transcription factors’ binding affinity to their specific corresponding regulatory motifs may significantly be correlated to human traits and diseases.

The SNPs which affect transcription factor binding affinity could influence the microRNA expression in several states including no effect (No change occurred in the TFBS for the original TFs) (neutral), gaining function (novel transcription factor attached to modified TFBS), and loss of function (original TFs cannot bind to its specific location). Part of a regulatory region to which no TF has previously been connected may connect some TFs; hence, novel TFBSs are successfully announced. Oliveira and et al. have shown that polymorphic C allele of IL-8-845 in promoter region can influence mRNA expression levels and disease risk (de Oliveira et al., 2015).

Sun et al. have announced that the changes in miRNA-binding sequencing sites have resulted in the loss of miRNA function (Sun et al., 2009). Therefore, SNPs in miRNAs can affect the function of RNA binding proteins. The interaction between RBP and miRNA plays a vital role in regulating the gene expression and impaired mRNA processing and expression, significantly linked to neurological disease. The miRNA polymorphism effect on altering its interaction with RBP in the pathogenesis of neurological diseases is still largely unknown. Thus, more in-depth studies may be needed to evaluate altered miRNA potential: RBP interaction as a diagnostic factor to predict disease progression. The list of SNPs occurring in miRNA gene promoters and RBP binding sites are presented in Table 4 and Supplementary Table 2.

The list of SNPs occurring in miRNA gene promoters and RBP binding sites are presented in Table 4 and Supplementary Table 2.

As a result, shows and we have expected, none of the SNPs were found in the GWAS catalog. Because GWAS is a whole genome sequencing technique and it determines SNPs in complementary DNA (cDNA), not in the non-coding areas, for example, intergenic and intronic loci. Ghanbari et al. have done the only GWAS study on microRNAs and AD. They indicated that miR-1229, by targeting SORL1, which are both expressed in the human brain, can cause Alzheimer’s disease (Table 1). They also found rs2291418 in the miR-1229 precursor to being significantly associated with Alzheimer’s disease, consistent with our data (Tables 2, 4; Ghanbari et al., 2016). rs2293481 in miR-106b is expression quantitative trait loci (eQTL) with *P*-value: 0.000004, Tissue Nerve Tibial, source: GTEx_V4 [Genotype-Tissue Expression (GTEx) consortium] (Table 3). It is revealed that tissue specificity is driven by context-dependent regulatory pathways, providing transcriptional regulation of tissue-specific processes (Sonawane et al., 2017).

Our study presents useful information on the possible impact of SNPs and different regulatory patterns on miRNA expression and function and provides valuable insights into the pathogenesis and development of AD. Finally, it seems that genetic variants could be the proper criteria for early detection of Alzheimer’s in the future.

## Conclusion

Briefly, following a deep screening of miRNAs that play a determining role in Alzheimer’s disease, several resources were implemented to annotate SNP’s functional effect in the miRNA gene. For a comprehensive study, we investigated various aspects of the mined SNPs effect on biogenesis and miRNA function, including pre-miRNA processing level, miRNA-target interaction, transcript level, and miRNA-RBPs interaction. This study theoretically provided a collection of candidate causal SNPs in different parts of the miRNA gene that could be considered for future practical study in Alzheimer’s disease management.

## Data Availability Statement

The original contributions presented in the study are included in the Supplementary Material, further inquiries can be directed to the corresponding author/s.

## Ethics Statement

This research was approved by ethics committee of the Hormozgan University of Medical Science (ethical cod: IR/HUMS.REC.270).

## Author Contributions

MM wrote the manuscript. MM, RM, HA, AN, and PM collected the data. PM revised the literature and contributed to the conception and design of the study. All authors contributed to the critical revision, edition, and final approval of the manuscript.

## Conflict of Interest

The authors declare that the research was conducted in the absence of any commercial or financial relationships that could be construed as a potential conflict of interest.

## Footnotes

1 http://www.cuilab.cn/hmdd

2 http://bioinfo.life.hust.edu.cn/guo_lab#!/

3 http://bioinfo.life.hust.edu.cn/miRNASNP#!/

4 http://asia.ensembl.org/index.html

5 ftp://ccg.vital-it.ch/snp2tfbs

6 https://genome.ucsc.edu/

7 http://ccg.vital-it.ch/snp2tfbs/

8 http://www.rbp-var.biols.ac.cn/

## References

[B1] Absalon S. Kochanek D. M. Raghavan V. Krichevsky A. M. (2013). MiR-26b, upregulated in Alzheimer’s disease, activates cell cycle entry, tau-phosphorylation, and apoptosis in postmitotic neurons. *J. Neurosci.* 33 14645–14659. 10.1523/jneurosci.1327-13.2013 24027266 PMC3810537

[B2] Andrés-León E. Cases I. Alonso S. Rojas A. M. (2017). Novel miRNA-mRNA interactions conserved in essential cancer pathways. *Sci. Rep.* 7 1–13. 10.1080/07391102.2020.1800511 28387377 PMC5384238

[B3] Banzhaf-Strathmann J. Benito E. May S. Arzberger T. Tahirovic S. Kretzschmar H. (2014). MicroRNA-125b induces tau hyperphosphorylation and cognitive deficits in Alzheimer’s disease. *EMBO J.* 33 1667–1680. 10.15252/embj.201387576 25001178 PMC4194100

[B4] Boissonneault V. Plante I. Rivest S. Provost P. (2009). MicroRNA-298 and microRNA-328 regulate expression of mouse beta-amyloid precursor protein-converting enzyme 1. *J. Biol. Chem.* 284 1971–1981. 10.1074/jbc.m807530200 18986979 PMC2908704

[B5] Boutz P. L. Chawla G. Stoilov P. Black D. L. (2007). MicroRNAs regulate the expression of the alternative splicing factor nPTB during muscle development. *Genes Dev.* 21 71–84. 10.1101/gad.1500707 17210790 PMC1759902

[B6] Buroker N. Ning X. Li K. Zhou Z. Cen W. (2015). SNPs, Linkage Disequilibrium and Transcriptional Factor Binding Sites Associated with Acute Mountain Sickness among Han Chinese at the Qinghai-Tibetan Plateau. *Int. J. Genomic Med.* 3:120.

[B7] Chan S. L. Huppertz I. Yao C. Weng L. Moresco J. J. Yates J. R. (2014). CPSF30 and Wdr33 directly bind to AAUAAA in mammalian mRNA 3′ processing. *Genes Dev.* 28 2370–2380. 10.1101/gad.250993.114 25301780 PMC4215182

[B8] Chen W. Wu L. Hu Y. Jiang L. Liang N. Chen J. (2020). MicroRNA-107 Ameliorates Damage in a Cell Model of Alzheimer’s Disease by Mediating the FGF7/FGFR2/PI3K/Akt Pathway. *J. Mol. Neurosci.* 70 1589–1597. 32472396 10.1007/s12031-020-01600-0

[B9] Cole S. L. Vassar R. (2007). The Alzheimer’s disease β-secretase enzyme, BACE1. *Mol. Neurodegenerat.* 2:22. 10.1186/1750-1326-2-22 18005427 PMC2211305

[B10] Croce N. Gelfo F. Ciotti M. T. Federici G. Caltagirone C. Bernardini S. (2013). NPY modulates miR-30a-5p and BDNF in opposite direction in an in vitro model of Alzheimer disease: a possible role in neuroprotection? *Mol. Cell Biochem.* 376 189–195. 10.1007/s11010-013-1567-0 23358924

[B11] de Oliveira J. G. Rossi A. F. T. Nizato D. M. Cadamuro A. C. T. Jorge Y. C. Valsechi M. C. (2015). Influence of functional polymorphisms in TNF-α, IL-8, and IL-10 cytokine genes on mRNA expression levels and risk of gastric cancer. *Tumor Biol.* 36 9159–9170. 10.1007/s13277-015-3593-x 26088449

[B12] Delay C. Calon F. Mathews P. Hébert S. S. (2011). Alzheimer-specific variants in the 3′UTR of Amyloid precursor protein affect microRNA function. *Mol. Neurodegenerat.* 6:70. 10.1186/1750-1326-6-70 21982160 PMC3195754

[B13] Dickson J. R. Kruse C. Montagna D. R. Finsen B. Wolfe M. S. (2013). Alternative polyadenylation and miR-34 family members regulate tau expression. *J. Neurochem.* 127 739–749. 24032460 10.1111/jnc.12437 PMC3859707

[B14] Dong H. Lei J. Ding L. Wen Y. Ju H. Zhang X. (2013). MicroRNA: function, detection, and bioanalysis. *Chem. Rev.* 113 6207–6233. 10.1021/cr300362f 23697835

[B15] Fang M. Wang J. Zhang X. Geng Y. Hu Z. Rudd J. A. (2012). The miR-124 regulates the expression of BACE1/beta-secretase correlated with cell death in Alzheimer’s disease. *Toxicol. Lett.* 209 94–105. 10.1016/j.toxlet.2011.11.032 22178568

[B16] Femminella G. D. Ferrara N. Rengo G. (2015). The emerging role of microRNAs in Alzheimer’s disease. *Front. Physiol.* 6:40. 10.3389/fphys.2015.00040 25729367 PMC4325581

[B17] Geekiyanage H. Chan C. (2011). MicroRNA-137/181c regulates serine palmitoyltransferase and in turn amyloid beta, novel targets in sporadic Alzheimer’s disease. *J. Neurosci.* 31 14820–14830. 10.1523/jneurosci.3883-11.2011 21994399 PMC3200297

[B18] Georges M. Coppieters W. Charlier C. (2007). Polymorphic miRNA-mediated gene regulation: contribution to phenotypic variation and disease. *Curr. Opin. Genet. Dev.* 17 166–176. 10.1016/j.gde.2007.04.005 17467975

[B19] Ghanbari M. Ikram M. A. de Looper H. W. J. Hofman A. Erkeland S. J. Franco O. H. (2016). Genome-wide identification of microRNA-related variants associated with risk of Alzheimer’s disease. *Sci. Rep.* 6:28387. 10.1038/srep28387 PMC4916596 27328823

[B20] Guo T. Feng Y. Liu Q. Yang X. Jiang T. Chen Y. (2014). MicroRNA-320a suppresses in GBM patients and modulates glioma cell functions by targeting IGF-1R. *Tumor Biol.* 35 11269–11275. 10.1007/s13277-014-2283-4 25117070

[B21] Hafner M. Landthaler M. Burger L. Khorshid M. Hausser J. Berninger P. (2010). Transcriptome-wide identification of RNA-binding protein and microRNA target sites by PAR-CLIP. *Cell* 141 129–141. 10.1016/j.cell.2010.03.009 20371350 PMC2861495

[B22] Höck J. Meister G. (2008). The Argonaute protein family. *Genome Biol.* 9:210. 10.1186/gb-2008-9-2-210 18304383 PMC2374724

[B23] Hu Y. Liu C.-M. Qi L. He T.-Z. Shi-Guo L. Hao C.-J. (2011). Two common SNPs in pri-miR-125a alter the mature miRNA expression and associate with recurrent pregnancy loss in a Han-Chinese population. *RNA Biol.* 8 861–872. 10.4161/rna.8.5.16034 21788734

[B24] Imperatore J. A. Then M. L. McDougal K. B. Mihailescu M. R. (2020). Characterization of a G-quadruplex structure in pre-miRNA-1229 and in its Alzheimer’s disease-associated variant rs2291418: implications for miRNA-1229 maturation. *Int. J. Mol. Sci.* 21:767. 10.3390/ijms21030767 31991575 PMC7037302

[B25] Inoue Y. Watanabe M. Inoue N. Kagawa T. Shibutani S. Otsu H. (2014). Associations of single nucleotide polymorphisms in precursor-microRNA (miR)-125a and the expression of mature mi R-125a with the development and prognosis of autoimmune thyroid diseases. *Clin. Exp. Immunol.* 178 229–235. 10.1111/cei.12410 24990808 PMC4233372

[B26] John B. Enright A. J. Aravin A. Tuschl T. Sander C. Marks D. S. (2004). Human microRNA targets. *PLoS Biol* 2:e363. 10.1371/journal.pbio.0020363 15502875 PMC521178

[B27] Jonsson T. Atwal J. K. Steinberg S. Snaedal J. Jonsson P. V. Bjornsson S. (2012). A mutation in APP protects against Alzheimer’s disease and age-related cognitive decline. *Nature* 488 96–99. 22801501 10.1038/nature11283

[B28] Kim J. Yoon H. Horie T. Burchett J. M. Restivo J. L. Rotllan N. (2015). microRNA-33 Regulates ApoE Lipidation and Amyloid-beta Metabolism in the Brain. *J. Neurosci.* 35 14717–14726. 10.1523/jneurosci.2053-15.2015 26538644 PMC4635126

[B29] Kim W. Noh H. Lee Y. Jeon J. Shanmugavadivu A. McPhie D. L. (2016). MiR-126 Regulates Growth Factor Activities and Vulnerability to Toxic Insult in Neurons. *Mol. Neurobiol.* 53 95–108. 10.1007/s12035-014-8989-x 25407931 PMC4437970

[B30] Kitagishi Y. Nakanishi A. Ogura Y. Matsuda S. (2014). Dietary regulation of PI3K/AKT/GSK-3β pathway in Alzheimer’s disease. *Alzheimer’s Res. Ther.* 6 1–7. 25031641 10.1186/alzrt265 PMC4075129

[B31] Lambert S. A. Jolma A. Campitelli L. F. Das P. K. Yin Y. Albu M. (2018). The human transcription factors. *Cell* 172 650–665. 29425488 10.1016/j.cell.2018.01.029 PMC12908702

[B32] Landi D. Gemignani F. Naccarati A. Pardini B. Vodicka P. Vodickova L. (2008). Polymorphisms within micro-RNA-binding sites and risk of sporadic colorectal cancer. *Carcinogenesis* 29 579–584. 10.1093/carcin/bgm304 18192692

[B33] Li L. Xu Y. Zhao M. Gao Z. (2020). Neuro-protective roles of long non-coding RNA MALAT1 in Alzheimer’s disease with the involvement of the microRNA-30b/CNR1 network and the following PI3K/AKT activation. *Exp. Mol. Pathol.* 117:104545. 10.1016/j.yexmp.2020.104545 32976819

[B34] Li X. Wang S.-W. Xi-Ling L. Yu F.-Y. Cong H.-M. (2020). Knockdown of long non-coding RNA TUG1 depresses apoptosis of hippocampal neurons in Alzheimer’s disease by elevating microRNA-15a and repressing ROCK1 expression. *Inflamm. Res.* 69 897–910. 10.1007/s00011-020-01364-8 32577774

[B35] Long J. M. Ray B. Lahiri D. K. (2014). MicroRNA-339-5p down-regulates protein expression of beta-site amyloid precursor protein-cleaving enzyme 1 (BACE1) in human primary brain cultures and is reduced in brain tissue specimens of Alzheimer disease subjects. *J. Biol. Chem.* 289 5184–5198. 10.1074/jbc.m113.518241 24352696 PMC3931075

[B36] Lukiw W. J. (2020). microRNA-146a Signaling in Alzheimer’s disease (AD) and prion disease (PrD). *Front. Neurol.* 11:462. 10.3389/fneur.2020.00462 32670176 PMC7331828

[B37] MacArthur J. Bowler E. Cerezo M. Gil L. Hall P. Hastings E. (2017). The new NHGRI-EBI Catalog of published genome-wide association studies (GWAS Catalog). *Nucleic Acids Res.* 45 D896–D901. 27899670 10.1093/nar/gkw1133 PMC5210590

[B38] Majewski J. Pastinen T. (2011). The study of eQTL variations by RNA-seq: from SNPs to phenotypes. *Trends Genet.* 27 72–79. 10.1016/j.tig.2010.10.006 21122937

[B39] Mao F. Xiao L. Li X. Liang J. Teng H. Cai W. (2016). RBP-Var: a database of functional variants involved in regulation mediated by RNA-binding proteins. *Nucleic Acids Res.* 44 D154–D163. 26635394 10.1093/nar/gkv1308 PMC4702914

[B40] Matsuda S. Nakagawa Y. Tsuji A. Kitagishi Y. Nakanishi A. Murai T. (2018). Implications of PI3K/AKT/PTEN signaling on superoxide dismutases expression and in the pathogenesis of Alzheimer’s disease. *Diseases* 6:28. 10.3390/diseases6020028 29677102 PMC6023281

[B41] Meng L. Li X.-Y. Shen L. Ji H.-F. (2020). Type 2 diabetes mellitus drugs for Alzheimer’s disease: current evidence and therapeutic opportunities. *Trends Mol. Med.* 26 597–614. 10.1016/j.molmed.2020.02.002 32470386

[B42] Mirzaii-Fini F. Dowlati M. A. Dehghani Ashkezari M. Kouchaki E. (2018). Investigating the association of Val/Met polymorphism of the BDNF gene with the incidence of disease in patients with Alzheimer and comparison with healthy elderly people in Iran. *FEYZ J. Kashan Univ. Med. Sci.* 22 617–623.

[B43] Moncini S. Lunghi M. Valmadre A. Grasso M. Del Vescovo V. Riva P. (2017). The miR-15/107 Family of microRNA Genes Regulates CDK5R1/p35 with Implications for Alzheimer’s Disease Pathogenesis. *Mol. Neurobiol.* 54 4329–4342. 10.1007/s12035-016-0002-4 27343180

[B44] Mullan M. Crawford F. Axelman K. Houlden H. Lilius L. Winblad B. (1992). A pathogenic mutation for probable Alzheimer’s disease in the APP gene at the N–terminus of β–amyloid. *Nature Genet.* 1 345–347. 10.1038/ng0892-345 1302033

[B45] Muller M. Kuiperij H. B. Claassen J. A. Kusters B. Verbeek M. M. (2014). MicroRNAs in Alzheimer’s disease: differential expression in hippocampus and cell-free cerebrospinal fluid. *Neurobiol. Aging* 35 152–158. 10.1016/j.neurobiolaging.2013.07.005 23962497

[B46] Pereira P. A. Tomas J. F. Queiroz J. A. Figueiras A. R. Sousa F. (2016). Recombinant pre-miR-29b for Alzheimer s disease therapeutics. *Sci. Rep.* 6:19946. 10.1038/srep19946 PMC4730146 26818210

[B47] Podhorna J. Winter N. Zoebelein H. Perkins T. Walda S. (2020). Alzheimer’s diagnosis: real-world physician behavior across countries. *Adv. Ther.* 37 883–893. 10.1007/s12325-019-01212-0 31933051 PMC7004426

[B48] Reddy P. H. Tonk S. Kumar S. Vijayan M. Kandimalla R. Kuruva C. S. (2017). A critical evaluation of neuroprotective and neurodegenerative MicroRNAs in Alzheimer’s disease. *Biochem. Biophys. Res. Commun.* 483 1156–1165. 10.1016/j.bbrc.2016.08.067 27524239 PMC5343756

[B49] Rockman M. V. Kruglyak L. (2006). Genetics of global gene expression. *Nat. Rev. Genet.* 7 862–872. 10.1038/nrg1964 17047685

[B50] Roy J. Mallick B. (2017). Altered gene expression in late-onset Alzheimer’s disease due to SNPs within 3′ UTR microRNA response elements. *Genomics* 109 177–185. 10.1016/j.ygeno.2017.02.006 28286146

[B51] Santa-Maria I. Alaniz M. E. Renwick N. Cela C. Fulga T. A. Van Vactor D. (2015). Dysregulation of microRNA-219 promotes neurodegeneration through post-transcriptional regulation of tau. *J. Clin. Invest.* 125 681–686. 10.1172/jci78421 25574843 PMC4319412

[B52] Sarkar S. Jun S. Rellick S. Quintana D. D. Cavendish J. Z. Simpkins J. W. (2016). Expression of microRNA-34a in Alzheimer’s disease brain targets genes linked to synaptic plasticity, energy metabolism, and resting state network activity. *Brain Res.* 1646 139–151. 10.1016/j.brainres.2016.05.026 27235866 PMC4975975

[B53] Sethi P. Lukiw W. J. (2009). Micro-RNA abundance and stability in human brain: specific alterations in Alzheimer’s disease temporal lobe neocortex. *Neurosci. Lett.* 459 100–104. 10.1016/j.neulet.2009.04.052 19406203

[B54] Shal B. Ding W. Ali H. Kim Y. S. Khan S. (2018). Anti-neuroinflammatory potential of natural products in attenuation of Alzheimer’s disease. *Front. Pharmacol.* 9:548. 10.3389/fphar.2018.00548 29896105 PMC5986949

[B55] Sonawane A. R. Platig J. Fagny M. Chen C.-Y. Paulson J. N. Lopes-Ramos C. M. (2017). Understanding tissue-specific gene regulation. *Cell Rep.* 21 1077–1088. 29069589 10.1016/j.celrep.2017.10.001 PMC5828531

[B56] Souza V. C. Morais G. S. Jr. Henriques A. D. Machado-Silva W. Perez D. I. V. Brito C. J. (2020). Whole-blood levels of MicroRNA-9 are decreased in patients with late-onset Alzheimer disease. *Am. J. Alzheimer’s Dis. Dementias* 35:1533317520911573. 10.1177/1533317520911573 PMC10623914 32301334

[B57] Starega-Roslan J. Witkos T. M. Galka-Marciniak P. Krzyzosiak W. J. (2015). Sequence features of Drosha and Dicer cleavage sites affect the complexity of isomiRs. *Int. J. Mol. Sci.* 16 8110–8127. 10.3390/ijms16048110 25867481 PMC4425070

[B58] Sun G. Yan J. Noltner K. Feng J. Li H. Sarkis D. A. (2009). SNPs in human miRNA genes affect biogenesis and function. *RNA* 15 1640–1651. 10.1261/rna.1560209 19617315 PMC2743066

[B59] Tian N. Cao Z. Zhang Y. (2014). MiR-206 decreases brain-derived neurotrophic factor levels in a transgenic mouse model of Alzheimer’s disease. *Neurosci. Bull.* 30 191–197. 10.1007/s12264-013-1419-7 24604632 PMC5562663

[B60] Tiribuzi R. Crispoltoni L. Porcellati S. Di Lullo M. Florenzano F. Pirro M. (2014). miR128 up-regulation correlates with impaired amyloid β (1-42) degradation in monocytes from patients with sporadic Alzheimer’s disease. *Neurobiol. Aging* 35 345–356. 10.1016/j.neurobiolaging.2013.08.003 24064186

[B61] Treiber T. Treiber N. Plessmann U. Harlander S. Daiß J.-L. Eichner N. (2017). A compendium of RNA-binding proteins that regulate microRNA biogenesis. *Mol. Cell* 66:270.–284. 28431233 10.1016/j.molcel.2017.03.014

[B62] Van Kouwenhove M. Kedde M. Agami R. (2011). MicroRNA regulation by RNA-binding proteins and its implications for cancer. *Nat. Rev. Cancer* 11 644–656. 10.1038/nrc3107 21822212

[B63] Vilardo E. Barbato C. Ciotti M. Cogoni C. Ruberti F. (2010). MicroRNA-101 regulates amyloid precursor protein expression in hippocampal neurons. *J. Biol. Chem.* 285 18344–18351. 10.1074/jbc.m110.112664 20395292 PMC2881760

[B64] Wahid F. Shehzad A. Khan T. Kim Y. Y. (2010). MicroRNAs: synthesis, mechanism, function, and recent clinical trials. *Biochim. Biophys. Acta* 1803 1231–1243. 10.1016/j.bbamcr.2010.06.013 20619301

[B65] Wang J. Chen C. Zhang Y. (2020). An investigation of microRNA-103 and microRNA-107 as potential blood-based biomarkers for disease risk and progression of Alzheimer’s disease. *J. Clin. Lab. Anal.* 34:e23006. 10.1002/jcla.23006 PMC6977154 31420923

[B66] Wang L. Liu J. Wang Q. Jiang H. Li Z. Liu R. (2019). MicroRNA-200a-3p mediates neuroprotection in Alzheimer-related deficits and attenuates amyloid-beta overproduction and tau hyperphosphorylation via co-regulating BACE1 and PRKACB. *Front. Pharmacol.* 10:806. 10.3389/fphar.2019.00806 31379578 PMC6658613

[B67] Weinberg R. B. Mufson E. J. Counts S. E. (2015). Evidence for a neuroprotective microRNA pathway in amnestic mild cognitive impairment. *Front. Neurosci.* 9:430. 10.3389/fnins.2015.00430 26594146 PMC4633499

[B68] West M. A. Kim K. Kliebenstein D. J. Van Leeuwen H. Michelmore R. W. Doerge R. (2007). Global eQTL mapping reveals the complex genetic architecture of transcript-level variation in Arabidopsis. *Genetics* 175 1441–1450. 10.1534/genetics.106.064972 17179097 PMC1840073

[B69] Witkos T. M. Koscianska E. Krzyzosiak W. J. (2011). Practical aspects of microRNA target prediction. *Curr. Mol. Med.* 11 93–109. 10.2174/156652411794859250 21342132 PMC3182075

[B70] Wong H. K. Veremeyko T. Patel N. Lemere C. A. Walsh D. M. Esau C. (2013). De-repression of FOXO3a death axis by microRNA-132 and -212 causes neuronal apoptosis in Alzheimer’s disease. *Hum. Mol. Genet.* 22 3077–3092. 10.1093/hmg/ddt164 23585551 PMC13387022

[B71] Xie G.-Y. Xia M. Miao Y.-R. Luo M. Zhang Q. Guo A.-Y. (2020). FFLtool: a web server for transcription factor and miRNA feed forward loop analysis in human. *Bioinformatics* 36 2605–2607. 10.1093/bioinformatics/btz929 31830251

[B72] Xu Y. Li L. Xiang X. Wang H. Cai W. Xie J. (2013). Three common functional polymorphisms in microRNA encoding genes in the susceptibility to hepatocellular carcinoma: a systematic review and meta-analysis. *Gene* 527 584–593. 10.1016/j.gene.2013.05.085 23791656

[B73] Yan R. Vassar R. (2014). Targeting the β secretase BACE1 for Alzheimer’s disease therapy. *Lancet Neurol.* 13 319–329. 10.1016/s1474-4422(13)70276-x 24556009 PMC4086426

[B74] Yang G. Song Y. Zhou X. Deng Y. Liu T. Weng G. (2015). MicroRNA-29c targets beta-site amyloid precursor protein-cleaving enzyme 1 and has a neuroprotective role in vitro and in vivo. *Mol. Med. Rep.* 12 3081–3088. 10.3892/mmr.2015.3728 25955795

[B75] Yang T. T. Liu C. G. Gao S. C. Zhang Y. Wang P. C. (2018). The Serum Exosome Derived MicroRNA− 135a,− 193b, and− 384 Were Potential Alzheimer’s Disease Biomarkers. *Biomed. Environ. Sci.* 31 87–96. 29606187 10.3967/bes2018.011

[B76] Zhang B. Chen C. F. Wang A. H. Lin Q. F. (2015). MiR-16 regulates cell death in Alzheimer’s disease by targeting amyloid precursor protein. *Eur. Rev. Med. Pharmacol. Sci.* 19 4020–4027. 26592823

[B77] Zhang C. Lu J. Liu B. Cui Q. Wang Y. (2016). Primate-specific miR-603 is implicated in the risk and pathogenesis of Alzheimer’s disease. *Aging* 8 272–290. 10.18632/aging.100887 26856603 PMC4789582

[B78] Zhang R. Zhang Q. Niu J. Lu K. Xie B. Cui D. (2014). Screening of microRNAs associated with Alzheimer’s disease using oxidative stress cell model and different strains of senescence accelerated mice. *J. Neurol. Sci.* 338 57–64. 10.1016/j.jns.2013.12.017 24423585

[B79] Zhang Y. Bai R. Liu C. Ma C. Chen X. Yang J. (2019). MicroRNA single-nucleotide polymorphisms and diabetes mellitus: A comprehensive review. *Clin. Genet.* 95 451–461. 30536647 10.1111/cge.13491

[B80] Zhang Y. Xing H. Guo S. Zheng Z. Wang H. Xu D. (2016). MicroRNA-135b has a neuroprotective role via targeting of beta-site APP-cleaving enzyme 1. *Exp. Ther. Med.* 12 809–814. 10.3892/etm.2016.3366 27446280 PMC4950157

[B81] Zhang Y.-W. Thompson R. Zhang H. Xu H. (2011). APP processing in Alzheimer’s disease. *Mol. Brain* 4 1–13. 21214928 10.1186/1756-6606-4-3 PMC3022812

[B82] Zhou B. Li L. Qiu X. Wu J. Xu L. Shao W. (2020). Long non-coding RNA ANRIL knockdown suppresses apoptosis and pro-inflammatory cytokines while enhancing neurite outgrowth via binding microRNA-125a in a cellular model of Alzheimer’s disease. *Mol. Med. Rep.* 22 1489–1497. 10.3892/mmr.2020.11203 32626959 PMC7339647

