# Supplementary material for: In silico Analysis of Polymorphisms in microRNAs Deregulated in Alzheimer Disease
Source: Front Neurosci. 2021 Mar 24;15:631852. doi: 10.3389/fnins.2021.631852 (PMC8024493; doi:10.3389/fnins.2021.631852)
Supplement: Supplementary file 1 [file Table_1.DOCX]

Supplementary:

Supplementary table 1: Structure of miRNAs which have been deregulated in Alzheimer.

| **miRNA** | **Accession** | **Genome position** | **Host gene** | **Mature miRNA** | **Cluster** |
| --- | --- | --- | --- | --- | --- |
|  |  |  |  |  |  |
| hsa-mir-101-2 | MI0000739 | chr9:4850297-4850375[+] | RCL1: Intronic | hsa-miR-101-2-5p | ------------------------------------ |
|  |  |  |  | hsa-miR-101-2-3p | ------------------------------------ |
| Has-mir-103 | MI0000109 | chr5:168560896-168560973(-) | PANK3 | hsa-miR-103-5p | 5kb:hsa-mir-103a-1, hsa-mir-103b-1  10kb:hsa-mir-103a-1, hsa-mir-103b-1 |
| hsa-mir-106a | [MI0000113](http://www.mirbase.org/cgi-bin/mirna_entry.pl?acc=MI0000113) | chrX:134170198- 134170278[-] | Intergenic | hsa-miR-103-3p | 5kb: hsa-mir-106a, hsa-mir-18b, hsa-mir-19b-2, hsa-mir-20b, hsa-mir-363, hsa-mir-92a-2 |
|  |  |  |  | [hsa-miR-106a-3p](http://www.mirbase.org/cgi-bin/mirna_entry.pl?acc=MIMAT0004517) | 10kb: hsa-mir-106a, hsa-mir-18b, hsa-mir-19b-2, hsa-mir-20b, hsa-mir-363, hsa-mir-92a-2 |
| hsa-mir-106b | MI0000734 | chr7:100093993-100094074[-] | MCM7: Intronic | hsa-miR-106b-5p | 5kb: hsa-mir-106b, hsa-mir-25, hsa-mir-93 |
|  |  |  |  | hsa-miR-106b-3p | 10kb: hsa-mir-106b, hsa-mir-25, hsa-mir-93 |
| hsa-miR-107 | MI0000114 | chr10:89592747-89592827(-) | PANK1: Intronic | hsa-miR-107 | ------------------------------------ |
| hsa-mir-181a-1 | MI0000289 | chr1:198859044-198859153[-] | Intergenic | hsa-miR-181a-5p | 5kb: hsa-mir-181a-1, hsa-mir-181b-1 |
|  |  |  |  | hsa-miR-181a-3p | 10kb: hsa-mir-181a-1, hsa-mir-181b-1 |
| hsa-mir-181a-2 | MI0000269 | chr9:124692442-124692551[+] | NR6A1: Intronic | hsa-miR-181a-5p | 5kb: hsa-mir-181a-2, hsa-mir-181b-2 |
|  |  |  |  | hsa-miR-181a-2-3p | 10kb: hsa-mir-181a-2, hsa-mir-181b-2 |
| hsa-mir-181b-1 | MI0000270 | chr1:198858873-198858982[-] | Intergenic | hsa-miR-181b-5p | 5kb: hsa-mir-181a-1, hsa-mir-181b-1 |
|  |  |  |  | hsa-miR-181b-3p | 10kb: hsa-mir-181a-1, hsa-mir-181b-1 |
| hsa-mir-181b-2 | MI0000683 | chr9:124693710-124693798[+] | NR6A1: Intronic | hsa-miR-181b-5p | 5kb: hsa-mir-181a-2, hsa-mir-181b-2 |
|  |  |  |  | hsa-miR-181b-2-3p | 10kb: hsa-mir-181a-2, hsa-mir-181b-2 |
| hsa-mir-181c | MI0000271 | chr19:13874699-13874808[+] | Intergenic | hsa-miR-181c-5p | 5kb: hsa-mir-181c, hsa-mir-181d |
|  |  |  |  | hsa-miR-181c-3p | 10kb: hsa-mir-181c, hsa-mir-181d |
| hsa-mir-181d | MI0003139 | chr19:13874875-13875011[+] | Intergenic | hsa-miR-181d-5p | 5kb: hsa-mir-181c, hsa-mir-181d |
|  |  |  |  | hsa-miR-181d-3p | 10kb: hsa-mir-181c, hsa-mir-181d |
| hsa-mir-1229 | MI0006319 | chr5:179798278-179798346[-] | MGAT4B: Intronic | hsa-miR-1229-5p | ----------------------------------------- |
|  |  |  |  | hsa-miR-1229-3p |  |
| hsa-mir-124 | MI0000443 | chr8:9903388-9903472[-] | Intergenic | hsa-miR-124-5p | ----------------------------------------- |
|  |  |  |  | hsa-miR-124-3p |  |
| hsa-mir-124-2 | MI0000444 | chr8:64379149-64379257[+] | Intergenic | hsa-miR-124-5p | ----------------------------------------- |
|  |  |  |  | hsa-miR-124-3p |  |
| hsa-mir-124-3 | MI0000445 | chr20:63178500-63178586[+] | Intergenic | hsa-miR-124-5p | ----------------------------------------- |
|  |  |  |  | hsa-miR-124-3p |  |
| hsa-mir-125a | MI0000469 | chr19:51693254-51693339[+] | SPACA6: Intronic | hsa-miR-125a-5p | 5kb: hsa-let-7e, hsa-mir-125a, hsa-mir-99b |
|  |  |  |  | hsa-miR-125a-3p | 10kb: hsa-let-7e, hsa-mir-125a, hsa-mir-99b |
| hsa-mir-125b-1 | MI0000446 | chr11:122099757-122099844[-] | Intergenic | hsa-miR-125b-5p | ----------------------------------------- |
|  |  |  |  | hsa-miR-125b-1-3p |  |
| hsa-mir-125b-2 | MI0000470 | chr21:16590237-16590325[+] | Intergenic | hsa-miR-125b-5p | ----------------------------------------- |
|  |  |  |  | hsa-miR-125b-2-3p |  |
| hsa-mir-126 | MI0000471 | chr9:136670602-136670686[+] | EGFL7: Intronic | hsa-miR-126-5p | ----------------------------------------- |
|  |  |  |  | hsa-miR-126-3p |  |
| hsa-mir-128-1 | MI0000447 | chr2:135665397-135665478[+] | R3HDM1: Intronic | hsa-miR-128-1-5p | ----------------------------------------- |
|  |  |  |  | hsa-miR-128-3p |  |
| hsa-mir-128-2 | MI0000727 | chr3:35744476-35744559[+] | ARPP21: Intronic | hsa-miR-128-3p | ----------------------------------------- |
|  |  |  |  | hsa-miR-128-2-5p |  |
| hsa-mir-130b | MI0000748 | chr22:21653304-21653385[+] | Intergenic | hsa-miR-130b-5p | 5kb: hsa-mir-130b, hsa-mir-301b |
|  |  |  |  | hsa-miR-130b-3p | 10kb: hsa-mir-130b, hsa-mir-301b |
| hsa-mir-132 | MI0000449 | chr17:2049908-2050008[-] | Intergenic | hsa-miR-132-5p | 5kb: hsa-mir-132, hsa-mir-212 |
|  |  |  |  | hsa-miR-132-3p | 10kb: hsa-mir-132, hsa-mir-212 |
| hsa-mir-135b | MI0000810 | chr1:205448302-205448398[-] | LEMD1; BLACAT1: Intronic | hsa-miR-135b-5p | ----------------------------------------- |
|  |  |  |  | hsa-miR-135b-3p |  |
| hsa-mir-137 | MI0000454 | chr1:98046070-98046171[-] | Intergenic | hsa-miR-137-5p | 5kb: hsa-mir-137, hsa-mir-2682 |
|  |  |  |  | hsa-miR-137-3p | 10kb: hsa-mir-137, hsa-mir-2682 |
| hsa-mir-146a | MI0000477 | chr5:160485352-160485450[+] | Intergenic | hsa-miR-146a-5p | ----------------------------------------- |
|  |  |  |  | hsa-miR-146a-3p |  |
| hsa-mir-146b | MI0003129 | MI0003129 | Intergenic | hsa-miR-146b-5p | ----------------------------------------- |
|  |  |  |  | hsa-miR-146b-3p |  |
| hsa-mir-15a | MI0000069 | chr13:50049119-50049201[-] | Intergenic | hsa-miR-15a-5p | 5kb: hsa-mir-15a, hsa-mir-16-1 |
|  |  |  |  | hsa-miR-15a-3p | 10kb: hsa-mir-15a, hsa-mir-16-1 |
| hsa-mir-15b | MI0000438 | chr3:160404588-160404685[+] | SMC4: Intronic | hsa-miR-15b-5p | 5kb: hsa-mir-15b, hsa-mir-16-2 |
|  |  |  |  | hsa-miR-15b-3p | 10kb: hsa-mir-15b, hsa-mir-16-2 |
| hsa-mir-16-1 | MI0000070 | chr13:50048973-50049061[-] | Intergenic | hsa-miR-16-5p | 5kb: hsa-mir-15a, hsa-mir-16-1 |
|  |  |  |  | hsa-miR-16-1-3p | 10kb: hsa-mir-15a, hsa-mir-16-1 |
| hsa-mir-16-2 | MI0000115 | chr3:160404745-160404825[+] | SMC4: Intronic | hsa-miR-16-5p | 5kb: hsa-mir-15b, hsa-mir-16-2 |
|  |  |  |  | hsa-miR-16-2-3p | 10kb: hsa-mir-15b, hsa-mir-16-2 |
| hsa-mir-188 | MI0000484 | chrX:50003503-50003588[+] | CLCN5: Intronic | hsa-miR-188-5p | 5kb: hsa-mir-188, hsa-mir-362, hsa-mir-500a, hsa-mir-500b, hsa-mir-501, hsa-mir-502, hsa-mir-532, hsa-mir-660 |
|  |  |  |  | hsa-miR-188-3p | 10kb: hsa-mir-188, hsa-mir-362, hsa-mir-500a, hsa-mir-500b, hsa-mir-501, hsa-mir-502, hsa-mir-532, hsa-mir-660 |
| hsa-mir-193b | MI0003137 | chr16:14303967-14304049[+] | Intergenic | hsa-miR-193b-5p | 10kb: hsa-mir-193b, hsa-mir-365a |
|  |  |  |  | hsa-miR-193b-3p |  |
| hsa-mir-20a | MI0000076 | chr13:91351065-91351135[+] | Intergenic | hsa-miR-20a-5p | 5kb: hsa-mir-17, hsa-mir-18a, hsa-mir-19a, hsa-mir-19b-1, hsa-mir-20a, hsa-mir-92a-1 |
|  |  |  |  | hsa-miR-20a-3p | 10kb: hsa-mir-17, hsa-mir-18a, hsa-mir-19a, hsa-mir-19b-1, hsa-mir-20a, hsa-mir-92a-1 |
| hsa-mir-206 | MI0000490 | chr6:52144349-52144434[+] | Intergenic | hsa-miR-206 | 5kb: hsa-mir-133b, hsa-mir-206 |
|  |  |  |  |  | 10kb: hsa-mir-133b, hsa-mir-206 |
| Has-mir-200 | MI0000737 | chr1:1167863-1167952[+] | Intergenic | hsa-miR-200a-5p hsa-miR-200a-3p | 5kb:hsa-mir-200a, hsa-mir-200b, hsa-mir-429  10kb:hsa-mir-200a, hsa-mir-200b, hsa-mir-429 |
| hsa-mir-212 | MI0000288 | chr17:2050271-2050380[-] | Intergenic | hsa-miR-212-5p | 5kb: hsa-mir-132, hsa-mir-212 |
|  |  |  |  | hsa-miR-212-3p | 10kb: hsa-mir-132, hsa-mir-212 |
| hsa-mir-219a-1 | MI0000296 | chr6:33207835-33207944[+] | Intergenic | hsa-miR-219a-5p | ----------------------------------------- |
|  |  |  |  | hsa-miR-219a-1-3p |  |
| hsa-mir-219a-2 | MI0000740 | chr9:128392618-128392714[-] | Intergenic | hsa-miR-219a-5p | 5kb: hsa-mir-219a-2, hsa-mir-219b |
|  |  |  |  | hsa-miR-219a-2-3p | 10kb: hsa-mir-219a-2, hsa-mir-219b |
| hsa-mir-219b | MI0017299 | chr9:128392621-128392708[+] | Intergenic | hsa-miR-219b-5p | 5kb: hsa-mir-219a-2, hsa-mir-219b |
|  |  |  |  | hsa-miR-219b-3p | 10kb: hsa-mir-219a-2, hsa-mir-219b |
| hsa-mir-23a | MI0000079 | chr19:13836587-13836659[-] | Intergenic | hsa-miR-23a-5p | 5kb: hsa-mir-23a, hsa-mir-24-2, hsa-mir-27a |
|  |  |  |  | hsa-miR-23a-3p | 10kb: hsa-mir-23a, hsa-mir-24-2, hsa-mir-27a |
| hsa-mir-23b | MI0000439 | chr9:95085208-95085304[+] | AOPEP: Intronic | hsa-miR-23b-5p | 5kb: hsa-mir-23b, hsa-mir-24-1, hsa-mir-27b, hsa-mir-3074 |
|  |  |  |  | hsa-miR-23b-3p | 10kb: hsa-mir-23b, hsa-mir-24-1, hsa-mir-27b, hsa-mir-3074 |
| hsa-mir-26b | MI0000084 | chr2:218402646-218402722[+] | CTDSP1: Intronic | hsa-miR-26b-5p | ----------------------------------------- |
|  |  |  |  | hsa-miR-26b-3p |  |
| hsa-mir-29a | MI0000087 | chr7:130876747-130876810[-] | Intergenic | hsa-miR-29a-5p | 5kb: hsa-mir-29a, hsa-mir-29b-1 |
|  |  |  |  | hsa-miR-29a-3p | 10kb: hsa-mir-29a, hsa-mir-29b-1 |
| hsa-mir-29b-1 | MI0000105 | chr7:130877459-130877539[-] | Intergenic | hsa-miR-29b-1-5p | 5kb: hsa-mir-29a, hsa-mir-29b-1 |
|  |  |  |  | hsa-miR-29b-3p | 10kb: hsa-mir-29a, hsa-mir-29b-1 |
| hsa-mir-29b-2 | MI0000107 | chr1:207802443-207802523[-] | Intergenic | hsa-miR-29b-3p | 5kb: hsa-mir-29b-2, hsa-mir-29c |
|  |  |  |  | hsa-miR-29b-2-5p | 10kb: hsa-mir-29b-2, hsa-mir-29c |
| hsa-mir-29c | MI0000735 | chr1:207801852-207801939[-] | Intergenic | hsa-miR-29c-5p | 5kb: hsa-mir-29b-2, hsa-mir-29c |
|  |  |  |  | hsa-miR-29c-3p | 10kb: hsa-mir-29b-2, hsa-mir-29c |
| hsa-mir-298 | MI0005523 | chr20:58818226-58818313[-] | Intergenic | hsa-miR-298 | 5kb: hsa-mir-296, hsa-mir-298 |
|  |  |  |  |  | 10kb: hsa-mir-296, hsa-mir-298 |
| hsa-mir-30a | MI0000088 | chr6:71403551-71403621[-] | Intergenic | hsa-miR-30a-5p | ----------------------------------------- |
|  |  |  |  | hsa-miR-30a-3p |  |
| hsa-mir-33a | MI0000091 | chr22:41900944-41901012[+] | SREBF2: Intronic | hsa-miR-33a-5p | ----------------------------------------- |
|  |  |  |  | hsa-miR-33a-3p |  |
| hsa-mir-33b | MI0003646 | chr17:17813836-17813931[-] | SREBF1: Intronic | hsa-miR-33b-5p | 5kb: hsa-mir-33b, hsa-mir-6777 |
|  |  |  |  | hsa-miR-33b-3p | 10kb: hsa-mir-33b, hsa-mir-6777 |
| hsa-mir-339 | MI0000815 | chr7:1022933-1023026[-] | C7orf50: Intronic | hsa-miR-339-5p | ----------------------------------------- |
|  |  |  |  | hsa-miR-339-3p |  |
| hsa-mir-34a | MI0000268 | chr1:9151668-9151777[-] | Intergenic | hsa-miR-34a-5p | ----------------------------------------- |
|  |  |  |  | hsa-miR-34a-3p |  |
| hsa-mir-34b | MI0000742 | chr11:111512938-111513021[+] | Intergenic | hsa-miR-34b-5p | 5kb: hsa-mir-34b, hsa-mir-34c |
|  |  |  |  | hsa-miR-34b-3p | 10kb: hsa-mir-34b, hsa-mir-34c |
| hsa-mir-34c | MI0000743 | chr11:111513439-111513515[+] | Intergenic | hsa-miR-34c-5p | 5kb: hsa-mir-34b, hsa-mir-34c |
|  |  |  |  | hsa-miR-34c-3p | 10kb: hsa-mir-34b, hsa-mir-34c |
| hsa-mir-328 | MI0000804 | chr16:67202321-67202395[-] | ELMO3: Intronic | hsa-miR-328-5p | ----------------------------------------- |
|  |  |  |  | hsa-miR-328-3p |  |
| hsa-mir-455 | MI0003513 | chr9:114209434-114209529[+] | COL27A1: Intronic | hsa-miR-455-5p | ----------------------------------------- |
|  |  |  |  | hsa-miR-455-3p |  |
| hsa-mir-603 | MI0003616 | chr10:24275685-24275781[+] | KIAA1217: Intronic | hsa-miR-603 | ----------------------------------------- |
| hsa-mir-329-1 | MI0001725 | chr14:101026785-101026864[+] | Intergenic | hsa-miR-329-5p | 5kb: hsa-mir-1193, hsa-mir-1197, hsa-mir-299, hsa-mir-323a, hsa-mir-329-1, hsa-mir-329-2, hsa-mir-379, hsa-mir-380, hsa-mir-411, hsa-mir-494, hsa-mir-495, hsa-mir-543, hsa-mir-758 |
|  |  |  |  | hsa-miR-329-3p | 10kb:hsa-mir-1185-1, hsa-mir-1185-2, hsa-mir-1193, hsa-mir-1197, hsa-mir-134, hsa-mir-154, hsa-mir-299, hsa-mir-300, hsa-mir-323a, hsa-mir-323b, hsa-mir-329-1, hsa-mir-329-2, hsa-mir-369, hsa-mir-376a-1, hsa-mir-376a-2, hsa-mir-376b, hsa-mir-376c, hsa-mir-377, hsa-mir-379, hsa-mir-380, hsa-mir-381, hsa-mir-382, hsa-mir-409, hsa-mir-410, hsa-mir-411, hsa-mir-412, hsa-mir-485, hsa-mir-487a, hsa-mir-487b, hsa-mir-494, hsa-mir-495, hsa-mir-496, hsa-mir-539, hsa-mir-541, hsa-mir-543, hsa-mir-544a, hsa-mir-654, hsa-mir-655, hsa-mir-656, hsa-mir-668, hsa-mir-758, hsa-mir-889 |
| hsa-mir-329-2 | MI0001726 | chr14:101027100-101027183[+] | Intergenic | hsa-miR-329-5p | 5kb: hsa-mir-1193, hsa-mir-1197, hsa-mir-299, hsa-mir-323a, hsa-mir-329-1, hsa-mir-329-2, hsa-mir-379, hsa-mir-380, hsa-mir-411, hsa-mir-494, hsa-mir-495, hsa-mir-543, hsa-mir-758 |
|  |  |  |  | hsa-miR-329-3p | 10kb:hsa-mir-1185-1, hsa-mir-1185-2, hsa-mir-1193, hsa-mir-1197, hsa-mir-134, hsa-mir-154, hsa-mir-299, hsa-mir-300, hsa-mir-323a, hsa-mir-323b, hsa-mir-329-1, hsa-mir-329-2, hsa-mir-369, hsa-mir-376a-1, hsa-mir-376a-2, hsa-mir-376b, hsa-mir-376c, hsa-mir-377, hsa-mir-379, hsa-mir-380, hsa-mir-381, hsa-mir-382, hsa-mir-409, hsa-mir-410, hsa-mir-411, hsa-mir-412, hsa-mir-485, hsa-mir-487a, hsa-mir-487b, hsa-mir-494, hsa-mir-495, hsa-mir-496, hsa-mir-539, hsa-mir-541, hsa-mir-543, hsa-mir-544a, hsa-mir-654, hsa-mir-655, hsa-mir-656, hsa-mir-668, hsa-mir-758, hsa-mir-889 |
| hsa-mir-9-1 | MI0000466 | chr1:156420341-156420429[-] | Intergenic | hsa-miR-9-5p | ----------------------------------------- |
|  |  |  |  | hsa-miR-9-3p |  |
| hsa-mir-9-2 | MI0000467 | chr5:88666853-88666939[-] | Intergenic | hsa-miR-9-5p | ----------------------------------------- |
|  |  |  |  | hsa-miR-9-3p |  |
| hsa-mir-9-3 | MI0000468 | chr15:89368017-89368106[+] | Intergenic | hsa-miR-9-5p | ----------------------------------------- |
|  |  |  |  | hsa-miR-9-3p |  |
|  |  |  |  |  |  |

Supplementary table 2: SNPs in miRNAs in the current study and their impact on other miRNAs expression patterns performed by the RBP-Var database.

| **chromosome** | **SNV_position** | **rs_ID** | **function** | **Gene name** | **miRNA name** | **P_value** |
| --- | --- | --- | --- | --- | --- | --- |
| chr7 | 99691652 | rs72631827 | loss | MIR106B | hsa-miR-5739 | 0 |
| chr7 | 99691652 | rs72631827 | gain | MIR106B | hsa-miR-4428 | 0 |
| chr10 | 91352545 | rs199975460 | loss | MIR107 | hsa-miR-5581-3p | 0.522938 |
| chr5 | 179225292 | rs200647784 | loss | MIR1229 | hsa-miR-34a-5p | 0.523433 |
| chr5 | 179225292 | rs200647784 | loss | MIR1229 | hsa-miR-34c-5p | 0.523605 |
| chr5 | 179225292 | rs200647784 | loss | MIR1229 | hsa-miR-449a | 0.523099 |
| chr5 | 179225292 | rs200647784 | loss | MIR1229 | hsa-miR-449b-5p | 0.523045 |
| chr5 | 179225292 | rs200647784 | gain | MIR1229 | hsa-miR-885-3p | 0 |
| chr5 | 179225324 | rs2291418 | gain | MIR1229 | hsa-miR-4639-3p | 0 |
| chr1 | 205417483 | rs139405984 | loss | MIR135B | hsa-miR-1302 | 0 |
| chr1 | 205417483 | rs139405984 | loss | MIR135B | hsa-miR-3120-3p | 0 |
| chr1 | 205417483 | rs139405984 | loss | MIR135B | hsa-miR-4298 | 0 |
| chr1 | 205417483 | rs139405984 | gain | MIR135B | hsa-miR-30c-2-3p | 0.183328 |
| chr1 | 205417483 | rs139405984 | gain | MIR135B | hsa-miR-30c-1-3p | 0.033212 |
| chr13 | 50623143 | rs72631826 | loss | MIR16-1 | hsa-miR-620 | 0 |
| chr13 | 50623143 | rs72631826 | loss | MIR16-1 | hsa-miR-1270 | 0 |
| chr13 | 50623143 | rs72631826 | gain | MIR16-1 | hsa-miR-4667-5p | 0.314666 |
| chr13 | 50623143 | rs72631826 | gain | MIR16-1 | hsa-miR-4700-5p | 0.311273 |
| chr7 | 1062656 | rs72631831 | loss | MIR339 | hsa-miR-4632-3p | 0 |
| chr7 | 1062656 | rs72631831 | gain | MIR339 | hsa-miR-324-3p | 0.497137 |
| chr7 | 1062656 | rs72631831 | gain | MIR339 | hsa-miR-1913 | 0.497047 |
| chr7 | 1062626 | rs145196722 | gain | MIR339 | hsa-miR-3607-5p | 0.487323 |
| chr7 | 1062626 | rs145196722 | gain | MIR339 | hsa-miR-5589-3p | 0.521488 |
| chr7 | 1062656 | rs72631831 | loss | MIR339 | hsa-miR-4632-3p | 0 |
| chr7 | 1062656 | rs72631831 | gain | MIR339 | hsa-miR-324-3p | 0.497137 |
| chr7 | 1062656 | rs72631831 | gain | MIR339 | hsa-miR-1913 | 0.497047 |
| chr1 | 9211782 | rs72631823 | gain | MIR34A | hsa-miR-4795-3p | 0.564587 |
| chr1 | 9211802 | rs35301225 | gain | MIR34A | hsa-miR-488-5p | 0 |
| chr13 | 50623143 | rs72631826 | loss | MIR16-1 | hsa-miR-620 | 0 |
| chr13 | 50623143 | rs72631826 | loss | MIR16-1 | hsa-miR-1270 | 0 |
| chr13 | 50623143 | rs72631826 | gain | MIR16-1 | hsa-miR-4667-5p | 0.314666 |
| chr13 | 50623143 | rs72631826 | gain | MIR16-1 | hsa-miR-4700-5p | 0.311273 |

| Supplementary table 3: | | | |
| --- | --- | --- | --- |
| miRNA | |  | **RBP binding site at miRNA** |
| miR-101-2 | | rs138231885 | TTCAGGTAGATATGAGACTGAACTG (T/C) CCTTTT (PTBP1) |
|  |  |  | TCAGGTAGATATGAGACTGAACTG (T/C) CCTTTTT (WDR33) |
| miR-106b | | rs72631827 |  |
|  |  |  | TGGAGCAGCAAGTACCCACAGTGCGGTAGCA(C/A/T) GGAGAGGACCACTATCTGCACTGTCAGCACTTTAGC (DGCR8) |
|  |  |  | GGAGCAGCAAGTACCCACAGTGCGGTAGCA(C/A/T) GGAGAGGACCACTATCTGCACTGTCAGCACTTTAG (AGO2) |
|  |  |  | GGAGCAGCAAGTACCCACAGTGCGGTAGCA(C/A/T) GGAGAGGACCACTATCTGCACTGTCAGCACTTTAGCCC (AGO2) |
|  |  |  | GAGCAGCAAGTACCCACAGTGCGGTAGCA(C/A/T) GGAGAGGACCACTATCTGCACTGTCAGCACTTTAG (AGO2) |
|  |  |  | GAGCAGCAAGTACCCACAGTGCGGTAGCA(C/A/T) GGAGAGGACCACTATCTGCACTGTCAGCACTTTAGC (AGO1) |
|  |  |  | GAGCAGCAAGTACCCACAGTGCGGTAGCA(C/A/T) GGAGAGGACCACTATCTGCACTGTCAGCACTTTAGC (AGO3) |
|  |  |  | AGCAGCAAGTACCCACAGTGCGGTAGCA(C/A/T) GGAGAGGACCACTATCTGCACTGTCAGCACTTTA (AGO2) |
|  |  |  | GCAGCAAGTACCCACAGTGCGGTAGCA(C/A/T) GGAGAGGACCACTATCTGCACTGTCAGCACTTTA (AGO1) |
|  |  |  | CACAGTGCGGTAGCA(C/A/T) G (WDR33) |
|  |  |  | CA(C/A/T) GGAGAGGACCACTATCTGCACTGTCAGCACTTTAG (AGO2) |
| miR-1229-3p | | rs200647784 |  |
|  | |  | CCTGTGGGAGGGCAG(T/C)GGTGAGAG (AGO2) |
|  |  |  | CCTGTGGGAGGGCAG(T/C)GGTGAGAGG (AGO1) |
|  |  |  | CCTGTGGGAGGGCAG(T/C)GGTGAGAGG (AGO2) |
|  |  |  | CCTGTGGGAGGGCAG(T/C)GGTGAGAGGG (AGO1) |
|  |  |  | CTGTGGGAGGGCAG(T/C)GGTGAGAG (AGO2) |
|  |  |  | CTGTGGGAGGGCAG(T/C)GGTGAGAGG |
| miR-1229-3p | | rs2291418 |  |
|  | |  | CCCAC(G/A)CTCTCCCCCAAACCCTACCCAC (AGO1) |
|  |  |  | AC(G/A)CTCTCCCCCAAACCCTACCCAC (AGO2) |
|  | |  |  |
| miR-126 | | rs199992070 |  |
| miR-128 | | rs117812383 |  |
|  | |  | TTGGCCTTGTTCCTGAGCTGTTGGATTCGGGGCC(G/A) TAGCACTGTCTGAGA GGTTTACA (DGCR8) |
|  |  |  | GTTCCTGAGCTGTTGGATTCGGGGCC(G/A) TAGCACTGTCTGAGAGGTTTACA (DGCR8) |
|  |  |  | TCGGGGCC(G/A)TAGCACTGTCTGA (AGO1) |
|  |  |  | TCGGGGCC(G/A)TAGCACTGTCTGAGA (AGO2) |
|  |  |  | TCGGGGCC(G/A)TAGCACTGTCTGAG (AGO3) |
| miR-130b | | rs72631822 |  |
|  | |  | ACTCTTTCCCTGTTGCACTACTATAGGCC(G/A/T)CTGGGA (PTBP1) |
|  |  |  | CTCTTTCCCTGTTGCACTACTATAGGCC(G/A/T)CTGGGAAGCAGTGCAATGATGAAAGGG |
| rs140403670 | | rs140403670 |  |
|  | | | CTCTTTCCCTGTTGCACTACTATAGGCCGCTGGGAAGCAGTGCAATGATGAAAGG(G/A) (eIF4AIII) |
|  |  |  | GAAGCAGTGCAATGATGAAAGG(G/A)CATCGGTCAG (DGCR8) |
|  |  |  | AGCAGTGCAATGATGAAAGG(G/A)CATC (AGO2) |
|  |  |  | AGCAGTGCAATGATGAAAGG(G/A)CATCG (AGO2) |
|  |  |  | GCAGTGCAATGATGAAAGG(G/A)C (WDR33) |
|  |  |  | GCAGTGCAATGATGAAAGG(G/A)C (AGO1) |
|  |  |  | GCAGTGCAATGATGAAAGG(G/A)C (AGO2) |
|  |  |  | GCAGTGCAATGATGAAAGG(G/A)C (AGO3) |
|  |  |  | GCAGTGCAATGATGAAAGG(G/A)C (LIN28A) |
|  |  |  | GCAGTGCAATGATGAAAGG(G/A)C (LIN28B) |
|  |  |  | GCAGTGCAATGATGAAAGG(G/A)CATC (AGO1) |
|  |  |  | CAGTGCAATGATGAAAGG(G/A)C (FMR1) |
|  |  |  | CAGTGCAATGATGAAAGG(G/A)CAT (WDR33) |
|  |  |  | CAGTGCAATGATGAAAGG(G/A)CAT (FMR1) |
|  |  |  | CAGTGCAATGATGAAAGG(G/A)CATCGGTCA (DGCR8) |
| miR-135b | | rs139405984 | GTTTGGGA(C/G/T)AGCAATCACATAGGAATGAAAAGCCATA(AGO2) |
| miR-146b | | rs76149940 | CACCTGGCA(C/T) (PTBP1) |
| miR-16 | | rs72631826 |  |
|  | |  | CTTCAGCAGCACAGTTAATACTGG(A/C)GATAATT (AGO2) |
|  |  |  | CTTCAGCAGCACAGTTAATACTGG(A/G)GATAATTTTAGAATCTTAACGCCAATATTTACGTGCTGCTAA (AGO1) |
|  |  |  | CTTCAGCAGCACAGTTAATACTGG(A/G)GATAATTTTAGAATCTTAACGCCAATATTTACGTGCTGCTAAGGCAC (AGO2) |
|  |  |  | TTCAGCAGCACAGTTAATACTGG(A/G)G (AGO2) |
|  |  |  | TTCAGCAGCACAGTTAATACTGG(A/G)GATAATTTTAGAATCTTAACGCCAATATTTACGTGCTGCTAA (AGO2) |
|  |  |  | TCAGCAGCACAGTTAATACTGG(A/G)GATAATTTTAGAATCTTAACG (eIF4AIII) |
|  |  |  | CAGCACAGTTAATACTGG(A/G)GATAATTTTAGAATCTT (nSR100) |
|  |  |  | AGCACAGTTAATACTGG(A/G)GATA (PTBP1) |
|  |  |  | G(A/G)GATAATTTTAGAATCTTAACGCCAATATTTACGTGCTGCTAA (AGO2) |
| miR-16 | | rs72631826 |  |
|  | |  | CTTCAGCAGCACAGTTAATACTGG(A/G)GATAATT (AGO2) |
|  |  |  | CTTCAGCAGCACAGTTAATACTGG(A/G)GATAATTTTAGAATCTTAACGCCAA  TATTTACGTGCTGCTAA (AGO1) |
|  |  |  | CTTCAGCAGCACAGTTAATACTGG(A/G)GATAATTTTAGAATCTTAACGCCAA  TATTTACGTGCTGCTAAGGCAC (AGO2) |
|  |  |  | TTCAGCAGCACAGTTAATACTGG(A/G)G (AGO2) |
|  |  |  | TTCAGCAGCACAGTTAATACTGG(A/G)GATAATTTTAGAATCTTAACGCCAAT  ATTTACGTGCTGCTA (AGO2) |
|  |  |  | TTCAGCAGCACAGTTAATACTGG(A/G)GATAATTTTAGAATCTTAACGCCAAT  ATTTACGTGCTGCTAA (AGO1) |
|  |  |  | TCAGCAGCACAGTTAATACTGG(A/G)GATAATTTTAGAATCTTAACG (eIF4AIII) |
|  |  |  | CAGCACAGTTAATACTGG(A/G)GATAATTTTAGAATCTT (nSR100) |
|  |  |  | AGCACAGTTAATACTGG(A/G)GATA (PTBP1) |
|  |  |  | G(A/G)GATAATTTTAGAATCTTAACGCCAATATTTACGTGCTGCTAA (AGO2) |
| miR-188 | | rs186369276 |  |
|  | |  | ACATCCCTTGCATGGTGGA(G/T) (WDR33) |
|  |  |  | ACATCCCTTGCATGGTGGA(G/T)G (WDR33) |
|  |  |  | ACATCCCTTGCATGGTGGA(G/T)G (AGO2) |
|  |  |  | ACATCCCTTGCATGGTGGA(G/T)GG (WDR33) |
|  |  |  | ACATCCCTTGCATGGTGGA(G/T)GG (AGO2) |
|  |  |  | ACATCCCTTGCATGGTGGA(G/T)GG (AGO1) |
|  |  |  | ACATCCCTTGCATGGTGGA(G/T)GG (AGO4) |
|  |  |  | ACATCCCTTGCATGGTGGA(G/T)GGT (WDR33) |
|  |  |  | ACATCCCTTGCATGGTGGA(G/T)GGT (AGO2) |
|  |  |  | ACATCCCTTGCATGGTGGA(G/T)GGT (AGO1) |
|  |  |  | ACATCCCTTGCATGGTGGA(G/T)GGTG (AGO1) |
|  |  |  | ACATCCCTTGCATGGTGGA(G/T)GGTG (AGO3) |
|  |  |  | CATCCCTTGCATGGTGGA(G/T)G (FUS) |
|  |  |  | CATCCCTTGCATGGTGGA(G/T)GGTGAGCT (DGCR8) |
| miR-188 | | rs191840972 |  |
|  | |  | CCTCCCA(C/T)ATGCAGGGTTTGCA (WDR33) |
|  |  |  | CCTCCCA(C/T)ATGCAGGGTTTGCA (AGO2) |
|  |  |  | CCTCCCA(C/T)ATGCAGGGTTTGCA (AGO1) |
|  |  |  | CCTCCCA(C/T)ATGCAGGGTTTGCA (AGO3) |
| miR-193 | | rs60406007 | CCGAGGATGGGAGCTGAGG(G/T)CTGGGTCTTTGCGGGCGAGATGAGGGTGTCGGATCAACTGGCCTACAAAGTCCCAGTTCTCGGCCCCCGGGACCAGCGTCTTC (DGCR8) |
| miR-20a | | rs185831554 |  |
|  | |  | CTTTTATTGTGTCGATGTAGAATCTGCCTGGTCTATCTGATGTGACAGCTTCTGTAGCACTAAAGTGCTTATAGTGCAGGTAGTGTTTAG(T/G)TATCTACTGCATTATGAGCACTTAAAGTACTGCTAGCTGTAGAACTCCAGCTTCGGCCTGTCGCCCAATCAAACTGTCCTGTTACTGAACACTGTTCTATGGTTAGTTTTGCAGGTTTGCATCCAGCTGTGTGATATTCTGCTGTGCAATCCATGCAAAACTGACTGTGGTAGTGAAAAGTCTGTAGAAAAGTAAGGGAAACTCAAACCCCTTTCTACACAGGTTGGATCGGTTGCAATGCTGTGTTTCT (DGCR8) |
|  |  |  | TTATTGTGTCGATGTAGAATCTGCCTGGTCTATCTGATGTGACAGCTTCTGTAGCACTAAAGTGCTTATAGTGCAGGTAGTGTTTAG(T/G) (DGCR8) |
|  |  |  | AGCACTAAAGTGCTTATAGTGCAGGTAGTGTTTAG(T/G)TATCTACTGCATTATGAGCACTTAAAGTAC (AGO2) |
|  |  |  | CACTAAAGTGCTTATAGTGCAGGTAGTGTTTAG(T/G)TATCTACTGCATTATGAGCACTTAAAGTA (AGO2) |
|  |  |  | ACTAAAGTGCTTATAGTGCAGGTAGTGTTTAG(T/G)TATCTACTGCATTATGAGCACTTAAAGT (AGO2) |
|  |  |  | ACTAAAGTGCTTATAGTGCAGGTAGTGTTTAG(T/G)TATCTACTGCATTATGAGCACTTAAAGT (AGO1) |
|  |  |  | CTAAAGTGCTTATAGTGCAGGTAGTGTTTAG(T/G)TATCTACTGCATTATGAGCACTTAAAGT (AGO1) |
|  |  |  | GTTTAG(T/G)TATCT (TIAL1) |
|  |  |  | GTTTAG(T/G)TATCTACTGCATTATGAGCACTTAAAGT (AGO2) |
|  |  |  | TTTAG(T/G)T (TIA1) |
|  |  |  | TAG)T/G)TATCTACTGCATTATGAGCACTT (nSR100) |
|  |  |  | G(T/G)TATCTACTGCATTATGAGCACTTAAAGT (AGO2) |
|  |  |  | G(T/G)TATCTACTGCATTATGAGCACTTAAAGT (AGO1) |
|  |  |  | G(T/G)TATCTACTGCATTATGAGCACTTAAAGT (AGO4) |
|  |  |  | G(T/G)TATCTACTGCATTATGAGCACTTAAAGT (LIN28B) |
|  |  |  | G(T/G)TATCTACTGCATTATGAGCACTTAAAGTA (AGO3) |
| miR23b | | rs201848546 |  |
|  | |  | CAGCACGGGGTGGCGCTGCTCTCAG(G/A) (PTBP1) |
|  |  |  | GTGGCGCTGCTCTCAG(G/A)TGCTCTGGCTGCTTGGGTTCCTGGCATGCTGATTTGTGACT (DGCR8) |
| miR-26b | | rs188612260 |  |
|  | |  | CCACCCTGCCCGGGACCCAGTTCAAGTAATTCAGGATAGGTTGTGTG(C/T)T (DGCR8) |
|  |  |  | GTTCAAGTAATTCAGGATAGGTTGTGTG(C/T)T (AGO2) |
|  |  |  | GTTCAAGTAATTCAGGATAGGTTGTGTG(C/T)TGTCCAGCCTGTTCTCCATTACTTGGCTC (AGO2) |
| miR-26b | | rs565919718 | CCACCCTGCC(C/T)GGGACCCAGTTCAAGTAATTCAGGATAGGTTGTGTGCT (DGCR8) |
| miR-298 | | rs201036298 |  |
|  | |  | CTGGGAGAACCTCCC(T/G)GCTT (AGO3) |
|  |  |  | CTGGGAGAACCTCCC(T/G)GCTTCTGCTGAAGACCTGAG (PTBP1) |
| miR-30a | | rs149150037 |  |
|  |  | | AGGCAGCTGCAAACATCC(G/A/C)ACTGAAAGCCCAT (DGCR8) |
|  |  |  | AGGCAGCTGCAAACATCC(G/A/C)ACTGAAAGCCCATCTG (DGCR8) |
|  |  |  | CAGCTGCAAACATCC(G/A/C)ACTGAAAGCCCAT (AGO2) |
|  |  |  | AGCTGCAAACATCC(G/A/C)ACTGAAA (WDR33) |
|  |  |  | AGCTGCAAACATCC(G/A/C)ACTGAAAG (WDR33) |
|  |  |  | AGCTGCAAACATCC(G/A/C)ACTGAAAG (AGO1) |
|  |  |  | AGCTGCAAACATCC(G/A/C)ACTGAAAG (AGO2) |
|  |  |  | AGCTGCAAACATCC(G/A/C)ACTGAAAG (AGO3) |
|  |  |  | AGCTGCAAACATCC(G/A/C)ACTGAAAGC (AGO2) |
|  |  |  | AGCTGCAAACATCC(G/A/C)ACTGAAAGCCCATCTGTGGCTT (eIF4AIII) |
|  |  |  | GCTGCAAACATCC(G/A/C)ACTGAAAG (WDR33) |
|  |  |  | GCTGCAAACATCC(G/A/C)ACTGAAAG (AGO1) |
|  |  |  | GCTGCAAACATCC(G/A/C)ACTGAAAG (AGO4) |
|  |  |  | GCTGCAAACATCC(G/A/C)ACTGAAAG (AGO2) |
|  |  |  | TGCAAACATCC(G/A/C)ACTGAAAG (AGO2) |
|  |  | | GCAAACATCC(G/A/C)ACTGAAA (AGO2) |
| miR-30a | | rs190842689 |  |
|  | | | CTTCACAGCTTCCAGT(C/A/G/T)GAGGATGTTTACAGTCGCTCACTGTCAACAGCAAT ( DGCR8) |
|  |  |  | TTCACAGCTTCCAGT(C/A/G/T)GAGGATGTTTACA (WDR33) |
|  |  |  | TTCACAGCTTCCAGT(C/A/G/T)GAGGATGTTTACAGTCGCTCACTGTCAACAGCA (DGCR8) |
|  |  |  | CACAGCTTCCAGT(C/A/G/T)GAGGATGTTTACA (WDR33) |
|  |  |  | CACAGCTTCCAGT(C/A/G/T)GAGGATGTTTACA (AGO2) |
|  |  |  | CACAGCTTCCAGT(C/A/G/T)GAGGATGTTTACAG (DGCR8) |
|  |  |  | CACAGCTTCCAGT(C/A/G/T)GAGGATGTTTACAG (AGO2) |
|  |  |  | ACAGCTTCCAGT(C/A/G/T)GAGGATGTTTACA (AGO2) |
|  |  |  | ACAGCTTCCAGT(C/A/G/T)GAGGATGTTTACA (AGO1) |
|  |  |  | ACAGCTTCCAGT(C/A/G/T)GAGGATGTTTACA (AGO3) |
|  |  |  | ACAGCTTCCAGT(C/A/G/T)GAGGATGTTTACA (AGO4) |
|  |  |  | CAGCTTCCAGT(C/A/G/T)GAGGATGTTTAC (WDR33) |
|  |  |  | CAGCTTCCAGT(C/A/G/T)GAGGATGTTTACA (WDR33) |
|  |  |  | CAGCTTCCAGT(C/A/G/T)GAGGATGTTTACA (AGO2) |
|  |  |  | CAGCTTCCAGT(C/A/G/T)GAGGATGTTTACA ( LIN28A) |
|  |  |  | CAGCTTCCAGT(C/A/G/T)GAGGATGTTTACAG (WDR33) |
|  |  |  | CAGCTTCCAGT(C/A/G/T)GAGGATGTTTACAGTCGCTCACTGTCAACAGCA (eIF4AIII) |
|  |  |  | AGCTTCCAGT(C/A/G/T)GAGGATGTTT (WDR33) |
|  |  |  | AGCTTCCAGT(C/A/G/T)GAGGATGTTTACA (WDR33) |
|  |  |  | AGCTTCCAGT(C/A/G/T)GAGGATGTTTACA (PTBP1) |
|  |  |  | AGCTTCCAGT(C/A/G/T)GAGGATGTTTACA (PTBP1) |
|  |  |  | AGCTTCCAGT(C/A/G/T)GAGGATGTTTACA ( FXR1) |
|  |  |  | AGCTTCCAGT(C/A/G/T)GAGGATGTTTACA ( FMR1) |
|  |  |  | GCTTCCAGT(C/A/G/T)GAGGATGTTTACA (WDR33) |
|  |  |  | CTTCCAGT(C/A/G/T)GAGGATGTTTACA (WDR33) |
|  | | | TTCCAGT(C/A/G/T)GAGGATGTTTACA (FUS) |
|  | | | CCAGT(C/A/G/T)GAGGATGTTTACA (FUS) |
|  | | | CCAGT(C/A/G/T)GAGGATGTTTACA (AGO2) |
|  | | | AGT(C/A/G/T)GAGGATGTTTACA (WDR33) |
| miR-33 | | rs77809319 |  |
|  | |  | GGTGCATTGTAGTTGCATTGCATGTTCTGGTGGTACCCATGCA(A/G)TGTTTCCACAGTGCATCA (AGO1) |
|  |  |  | ACCCATGCA(A/G)TGTTTCCACAGTGCA (PTBP1) |
|  |  |  | CATGCA(A/G)TGTTTCCACAGTGCATCA (AGO2) |
|  |  |  | CATGCA(A/G)TGTTTCCACAGTGCATCA (AGO3) |
|  |  |  | ATGCA(A/G)TGTTTCCACAG (AGO2) |
|  |  |  | ATGCA(A/G)TGTTTCCACAGTGCATCA (AGO2) |
|  |  |  | GCA(A/G)TGTTTCCACAGTGCATCA (WDR33) |
| miR-339 | | rs72631831 | GCACACGTGAGCTCCTGGAGGACAGGGAGAGCGGC(C/T)GCCCCGCCCCTGCG (DGCR8) |
| miR-339 | | rs72631820 |  |
|  | |  | CGCCGGCTCTG(T/C)CGTCGAGGCGCTCACA (DGCR8) |
|  |  |  | CGCCGGCTCTG(T/C)CGTCGAGGCGCTCACAGGC (DGCR8) |
|  |  |  | GCCGGCTCTG(T/C)CGTCGAGGCGCTCACAGGCAGGCACACGTGAGCTCCTGGAGGACAGGGAGA (AGO2) |
|  |  |  | CCGGCTCTG(T/C)CGTCGAGGC (WDR33) |
|  |  |  | CCGGCTCTG(T/C)CGTCGAGGCGCT (WDR33) |
|  |  |  | CCGGCTCTG(T/C)CGTCGAGGCGCTC (WDR33) |
|  |  |  | CCGGCTCTG(T/C)CGTCGAGGCGCTCA (WDR33) |
|  |  |  | CCGGCTCTG(T/C)CGTCGAGGCGCTCA (AGO2) |
|  |  |  | CCGGCTCTG(T/C)CGTCGAGGCGCTCA (AGO3) |
|  |  |  | CCGGCTCTG(T/C)CGTCGAGGCGCTCACAG (AGO1) |
|  |  |  | CCGGCTCTG(T/C)CGTCGAGGCGCTCACAGG (AGO2) |
|  |  |  | CCGGCTCTG(T/C)CGTCGAGGCGCTCACAGGCAGGCACACGTGAGCTCCTGGAGGACAGGGAGA (AGO2) |
|  |  |  | GGCTCTG(T/C)CGTCGAGGCGCTCA (WDR33) |
|  |  |  | GGCTCTG(T/C)CGTCGAGGCGCTCA (AGO1) |
|  |  |  | GCTCTG(T/C)CGTCGAGGCGCTCA (AGO2) |
| miR-339 | | rs145196722 |  |
|  | |  | GCCGGCTCTGTCGTCGAGGCGCTCACAGGCAGGCACA(C/T)GTGAGCTCCTGGAGGACAGGGAGA (AGO2) |
|  |  |  | CCGGCTCTGTCGTCGAGGCGCTCACAGGCAGGCACA(C/T)GTGAGCTCCTGGAGGACAGGGAGA (AGO2) |
|  |  |  | CACAGGCAGGCACA(C/T)GTGAGCTCCTGGAGGACAGGGA (AGO2) |
|  |  |  | GGCACA(C/T)GTGAGCTCCTGGAGGACAGGGAGAG (DGCR8) |
|  |  |  | GCACA(C/T)GTGAGCTCCTGGAGGACAGGGAGAGCGGCCGCCCCGCCCCTGCG (DGCR8) |
|  |  |  | CACA(C/T)GTGAGCTCCTGGAGGACAGGGA (WDR33) |
|  |  |  | ACA(C/T)GTGAGCTCCTGGAGGACAGGGA (AGO1) |
|  |  |  | CA(C/T)GTGAGCTCCTGGAGGACAGGGA (WDR33) |
|  |  |  | CA(C/T)GTGAGCTCCTGGAGGACAGGGA (AGO1) |
|  |  |  | CA(C/T)GTGAGCTCCTGGAGGACAGGGA (AGO2) |
|  |  |  | CA(C/T)GTGAGCTCCTGGAGGACAGG (WDR33) |
|  |  |  | CA(C/T)GTGAGCTCCTGGAGGACAGG (AGO1) |
|  |  |  | CA(C/T)GTGAGCTCCTGGAGGACAGG (AGO2) |
|  |  |  | CA(C/T)GTGAGCTCCTGGAGGACAGG (AGO3) |
| miR-339-5p | | rs567174785 |  |
|  | |  | GCACACGTGAGCTCCTGGAGGACAGGGAGAGC(G/A)GCCGCCCCGCCCCTGCG (DGCR8) |
|  |  |  | GCACACGTGAGCTCCTGGAGGACAGGGAGAGC(G/A) (WDR33) |
| miR-34a | | rs201359809 |  |
|  | |  | GCA(C/G)TTCTAGGGCAGTATACTTGCTGATT (AGO2) |
|  |  |  | (C/G)ACTTCTAGGGCAGTATACTTGCTGATTGCTTCCTTACTATTGCTCACAACAACCAGCTAAGACACTGCCAAAG ( DGCR8) |
| miR-34a | | rs72631823 |  |
|  | |  | ACTTCTAGGGCAGTATACTTGCTGATTGCTTCCTTA(C/T)TATTGCTCACAACAACCAGCTAAGACACTGCCAAAG ( DGCR8) |
|  |  |  | TTCTAGGGCAGTATACTTGCTGATTGCTTCCTTA(C/T)TATTGCTCACAACAACCAGCTAAGACACTGCCAA (AGO1) |
|  |  |  | TTCTAGGGCAGTATACTTGCTGATTGCTTCCTTA(C/T)TATTGCTCACAACAACCAGCTAAGACACTGCCAAA (AGO2) |
|  |  |  | CTGATTGCTTCCTTA(C/T)TATTGCTCACAACAACCAGC ( nSR100) |
|  |  |  | CTGATTGCTTCCTTA(C/T)TATTGCTCACAACAACCAGCTAAGACACTGCCA (AGO2) |
| miR-34a | | rs35301225 |  |
|  | |  | ACTTCTAGGGCAGTATACTTGCTGATTGCTTCCTTACTATTGCTCACAACAACCAG(C/A/T)TAAGACACTGCCAAAG (DGCR8) |
|  |  |  | TTCTAGGGCAGTATACTTGCTGATTGCTTCCTTACTATTGCTCACAACAACCAG(C/A/T)TAAGACACTGCCAA (AGO1) |
|  |  |  | TTCTAGGGCAGTATACTTGCTGATTGCTTCCTTACTATTGCTCACAACAACCAGCTAAGACACTGCCAAA (AGO2) |
|  |  |  | CTGATTGCTTCCTTACTATTGCTCACAACAACCAG(C/A/T) (nSR100) |
|  |  |  | TACTATTGCTCACAACAACCAG(C/A/T)TAAGACACTGCCA (AGO2) |
|  |  |  | CTATTGCTCACAACAACCAG(C/A/T)TAAGACACTGCCAA (AGO2) |
|  |  |  | TTGCTCACAACAACCAG(C/A/T)TAAGACACTGCCA (AGO2) |
|  |  |  | TTGCTCACAACAACCAG(C/A/T)TAAGACACTGCCAAA (AGO2) |
|  |  |  | TCACAACAACCAG(C/A/T)TAAGACACTGCCA (WDR33) |
|  |  |  | TCACAACAACCAG(C/A/T)TAAGACACTGCCAAA (DGCR8) |
|  |  |  | ACAACAACCAG(C/A/T)TAAGACACTGCCA (PTBP1) |
|  |  |  | ACAACAACCAG(C/A/T)TAAGACACTGCCA (AGO1) |
|  |  |  | ACAACAACCAG(C/A/T)TAAGACACTGCCAA (AGO3) |
|  |  |  | CAACAACCAG(C/A/T)TAAGAC (WDR33) |
|  |  |  | CAACAACCAG(C/A/T)TAAGACACTGCC (WDR33) |
|  |  |  | CAACAACCAG(C/A/T)TAAGACACTGCCA (WDR33) |
|  |  |  | CAACAACCAG(C/A/T)TAAGACACTGCCA (FUS) |
|  |  |  | CAACAACCAG(C/A/T)TAAGACACTGCCA (AGO2) |
|  |  |  | CAACAACCAG(C/A/T)TAAGACACTGCCA (C22ORF28) |
|  |  |  | CAACAACCAG(C/A/T)TAAGACACTGCCA (AGO1) |
|  |  |  | CAACAACCAG(C/A/T)TAAGACACTGCCA (AGO4) |
|  |  |  | CAACAACCAG(C/A/T)TAAGACACTGCCAA (AGO2) |
|  |  |  | AACAACCAG(C/A/T)TAAGACACTGCCA ( WDR33) |
|  |  |  | AACAACCAG(C/A/T)TAAGACACTGCCA ( FUS) |
|  |  |  | ACAACCAG(C/A/T)TAAGACACTGCCA (WDR33) |
|  |  |  | ACAACCAG(C/A/T)TAAGACACTGCCA (FMR1) |
|  |  |  | ACAACCAG(C/A/T)TAAGACACTGCCA (AGO2) |
|  |  |  | ACAACCAG(C/A/T)TAAGACACTGCCA (FXR1) |
